# Re-entry in models of cardiac ventricular tissue with scar represented as a Gaussian random field

**DOI:** 10.3389/fphys.2024.1403545

**Published:** 2024-06-28

**Authors:** Richard H. Clayton, S. Sridhar

**Affiliations:** Insigneo Institute for in-silico Medicine and Department of Computer Science, University of Sheffield, Sheffield, United Kingdom

**Keywords:** cardiac electrophysiology, computer model, cardiac arrhythmia, human ventricles, fibrosis, Gaussian random field, re-entry

## Abstract

**Introduction:** Fibrotic scar in the heart is known to act as a substrate for arrhythmias. Regions of fibrotic scar are associated with slowed or blocked conduction of the action potential, but the detailed mechanisms of arrhythmia formation are not well characterised and this can limit the effective diagnosis and treatment of scar in patients. The aim of this computational study was to evaluate different representations of fibrotic scar in models of 2D 10 × 10 cm ventricular tissue, where the region of scar was defined by sampling a Gaussian random field with an adjustable length scale of between 1.25 and 10.0 mm.

**Methods:** Cellular electrophysiology was represented by the Ten Tusscher 2006 model for human ventricular cells. Fibrotic scar was represented as a spatially varying diffusion, with different models of the boundary between normal and fibrotic tissue. Dispersion of activation time and action potential duration (APD) dispersion was assessed in each sample by pacing at an S1 cycle length of 400 ms followed by a premature S2 beat with a coupling interval of 323 ms. Vulnerability to reentry was assessed with an aggressive pacing protocol. In all models, simulated fibrosis acted to delay activation, to increase the dispersion of APD, and to generate re-entry.

**Results:** A higher incidence of re-entry was observed in models with simulated fibrotic scar at shorter length scale, but the type of model used to represent fibrotic scar had a much bigger influence on the incidence of reentry.

**Discussion:** This study shows that in computational models of fibrotic scar the effects that lead to either block or propagation of the action potential are strongly influenced by the way that fibrotic scar is represented in the model, and so the results of computational studies involving fibrotic scar should be interpreted carefully.

## 1 Introduction

The heart is an electromechanical pump, where mechanical contraction is both initiated and synchronised by a propagating wave of electrical activation, the action potential. Action potentials originate in the heart’s natural pacemaker and propagate through the myocardium because cardiac myocytes are both electrically excitable and coupled through gap junctions. Abnormal slowing or block of action potential propagation can result in a cardiac arrhythmia. Arrhythmias sustained by re-entry, where an action potential continually propagates into recovering tissue, can be lethal if untreated (de Jong et al., 2011; Ciaccio et al., 2022).

Myocardial ischaemia and infarction are well known as an important substrate for initiating and sustaining re-entrant arrhythmias in the ventricles (Janse and Wit, 1989; Nguyen et al., 2014). A small surviving isthmus of electrically excitable tissue within the infarct region or border zone between infarct and normal myocardium can act as a re-entrant circuit (Anter et al., 2016; Pashakhanloo et al., 2018), and is a target for ablation to treat ventricular tachycardia (VT) in the human heart. Some simulation studies have shown that the structural heterogeneity of the infarct and border zone alone are sufficient to sustain re-entry when it is initiated by rapid pacing (Ten Tusscher and Panfilov, 2007; Engelman et al., 2010; Rutherford et al., 2012), while others have shown that functional remodelling may also play a role (Zahid et al., 2016; Costa et al., 2018). It is difficult to study properties of scar *in-vivo* (Anter et al., 2016), and studies of cultured tissue and cells may not be representative of the *in-vivo* setting (Nguyen et al., 2014). Computational models of infarct scar structure and function therefore have a role to play in identifying and understanding physiologically plausible mechanisms.

The task of modelling the structure of infarct and border zone is however challenging, and there is no consensus on the best way to represent the complex mixture of myocytes, fibroblasts, and collagen that compose the fibrotic scar (Calcagno et al., 2022; Simon-Chica et al., 2023). Several different approaches for modelling the structure of scar in cardiac tissue have been taken. A relatively straightforward approach to represent fibrotic scar is to designate randomly selected points within a finite difference grid as uncoupled and inexcitable, and this type of model reconstructs propagation slowing, block, and re-entry (Ten Tusscher and Panfilov, 2007; Alonso and Bär, 2013). Random removal of finite elements in models of atrial fibrosis (Roney et al., 2016), and representation of fibrotic clefts in models of diffuse ventricular fibrosis by setting some element edges to be electrically insulating (Mendonca Costa et al., 2014; Balaban et al., 2018), have also been effective in reconstructing re-entry mechanisms. More exotic approaches have used statistical models to represent different patterns and texture of tissue fibrosis (Clayton, 2018; Jakes et al., 2019; Nezlobinsky et al., 2021).

The functional effect of fibrotic scar on action potential conduction velocity and action potential duration (Amoni et al., 2023) is difficult to tease out from electrotonic effects within the altered structure (Connolly and Bishop, 2016). The electrophysiological behaviour of fibroblasts within tissue is not well understood (Simon-Chica et al., 2023), but approaches including explicit representation of the fibroblast domain using a model of fibroblast cellular electrophysiology (Sachse et al., 2009) and homogenisation of the myocyte/fibroblast mixture (Lawson et al., 2020; 2023) show promise.

The overall aims of the present study were therefore to:

• Construct a set of plausible structural models of infarct scar and border zone, with different texture and spatial scale to reflect both focal and diffuse fibrotic scar.
• Compare activation, recovery, and vulnerability to re-entry in different functional models of coupling between fibrotic scar and normal tissue.

## 2 Methods

Our starting point was to develop structural models of fibrotic scar representing a focal infarct surrounded by a border zone. We sought a plausible way to represent tissue with a central inexcitable region, with surrounding regions having a patchy border zone with focal and diffuse fibrosis. Evidence from tissue culture (Gaudesius et al., 2003) and experimental preparations (Ghouri et al., 2018) supports the idea of conduction slowing in regions of border zone and scar. However the distribution of scar regions within the border zone tends to be variable and irregular (Pashakhanloo et al., 2018; Amoni et al., 2023). We therefore based our approach on previous studies where spatial changes in the diffusion coefficient with different length scale are used to represent varying proportions of fibroblasts and myocytes (Jacquemet and Henriquez, 2008; Clayton, 2018).

Re-entry in ventricular myocardium is often sustained by retrograde conduction through a narrow isthmus of surviving tissue surrounded by regions of scar (Ciaccio et al., 2022). Ventricular myocardium is 3-dimensional, with a fibre-sheet structure that results in orthotropic propagation of the action potential (Clayton et al., 2011; Rutherford et al., 2012). In the present study we simplified this complex structure to a 2-dimensional (2D) tissue sheet. This approach enabled us to focus on different models of scar in a large number of samples and simulations, without the confounding effects of anisotropy, the more complex analysis of simulation output, and the additional computational burden of 3D simulations (Balaban et al., 2020).

### 2.1 Model of cell and tissue electrophysiology

Our model of a 2D ventricular tissue sheet had dimensions 10×10cm . Cellular electrophysiology was described by the Ten Tusscher 2006 (TNNP06) model for human ventricular myocytes (ten Tusscher and Panfilov, 2006), with parameters set for epicardial cells and steep APD restitution (parameter set four from the original paper). Tissue electrophysiology was described using the monodomain equation (Clayton et al., 2011), with no-flux boundary conditions at the edges. Different representations of a central infarct and border zone were imposed on this tissue model as described below.

The monodomain equation has a diffusion term to represent the spread of excitation and recovery, and a reaction term that represents local excitation and recovery Eq. 1. At a single location in 2D, the rate of change of transmembrane voltage is given by

$$\frac{\partial V\left(x,y,t\right)}{\partial t}=\nabla \cdot \left(D\left(x,y\right)\nabla V\left(x,y,t\right)\right)-\frac{I_{\mathrm{ion}}}{C_{m}},$$ (1)

where D(x,y) is a spatially varying diffusion coefficient for isotropic conduction, $I_{\mathrm{ion}}$ is local current flow through the cell membrane given by the cellular electrophysiology model, and $C_{m}$ is the specific cellular capacitance.

### 2.2 Model implementation and numerical methods

The cell and tissue electrophysiology model was implemented using our own finite difference code, which has been benchmarked against other codes (Niederer et al., 2011) (https://github.com/RichardClayton/VentricularFibrosis). This code solves the monodomain equation using explicit finite differences, with Rush Larsen approximation for the cellular electrophysiology model ODEs (Rush and Larsen, 1978), a space step of 0.25mm , a time step for the diffusion term of 0.05ms , and an adaptive time step for the reaction term between 0.001 and 0.1ms (Qu and Garfinkel, 1999). No-flux boundary conditions were imposed at the edges of the sheet; see below for details about how boundary conditions were applied to regions of simulated scar.

### 2.3 Structural representation of infarct and border zone

To produce a model of border zone and scar we used a Gaussian random field (GRF) to modulate tissue diffusion (Kroese and Botev, 2015). GRFs have a characteristic length scale, and are specified by a mean and a variance. A GRF can be sampled repeatedly to obtain a set of smoothly changing fields, each with an identical length scale, mean, and variance, but with a randomly generated pattern. In this study, we used different length scales to produce different textures to broadly represent diffuse and patchy fibrosis. We generated GRFs using circulant embedding with a squared exponential covariance function and a specified length scale (Kroese and Botev, 2015). Each GRF was then sampled to produce smoothly varying fields with fluctuations at specified length scales in a 2D tissue sheet as in a previous study (Clayton, 2018). The algorithm we used to generate GRFs was based on Matlab code provided on pages 376-377 of (Kroese and Botev, 2015), and is provided in the Supplementary Material.

**FIGURE 1:**
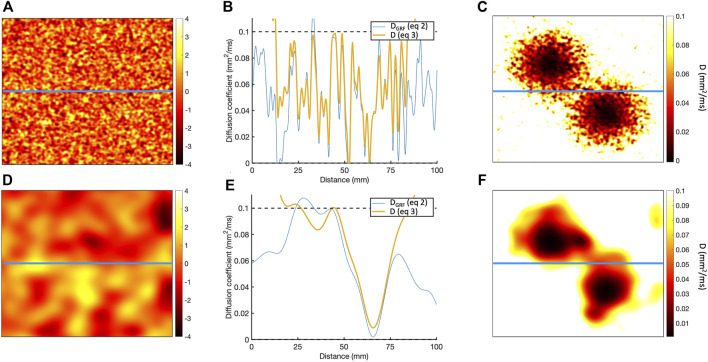
**(A)** Sample of Gaussian random field for length scale of 1.25 mm. **(B)** Horizontal cross-section of Gaussian random field along the blue line in A, showing the $D_{\mathrm{GRF}}$ obtained using Eq. 2 in blue, and D obtained using Eq. 3 in orange. **(C)** Resulting diffusion field D . **(D–F)** Corresponding plots for length scale of 20 mm.

We sampled GRFs at length scales of 1.25,2.5,5.0 , and 10.0mm . Each GRF sample provided a different pattern of fibrosis with the specified length scale, and by obtaining 20 samples at each length scale we aimed to capture a representative range of behaviours. At a length scale of 1.25mm the fluctuations in the GRF are broadly consistent with diffuse fibrosis (Figures 1A–C), but at longer length scale the fluctuations resembled more patchy fibrosis (Figures 1D–F) (de Jong et al., 2011). The raw GRF was then transformed to a diffusion field $D_{\mathrm{GRF}}$ that extended across the entire tissue sheet:

$$D_{\mathrm{GRF}}\left(x,y\right)=D_{\max}\frac{\left(GRF\left(x,y\right)+2.0\right)}{4.0},$$ (2)

where $D_{\max}$ was the upper limit of the diffusion coefficient, set to $0.1\;mm^{2}/ms$ . Each GRF sample had a mean of zero, and a standard deviation of 1.0, so the diffusion field $D_{\mathrm{GRF}}$ varied predominantly between zero and $D_{\max}$ .

To represent a 2D scar with a central isthmus, $D_{\mathrm{GRF}}$ was smoothed using sigmoid functions to give a field representing the diffusion coefficient, with two regions of scar located on the diagonal, each with a central zone representing the infarct and a surrounding region representing the infarct border zone. The first was centred at one-third of the sheet width, and the second at two-thirds of the sheet width. At each location (x,y) in the tissue sheet the diffusion coefficient with units $mm^{2}/ms$ was given by:

$$\begin{array}{l} D\left(x,y\right)=\frac{D_{\max}}{\left(1.0+exp\left(-0.075\left(r_{1}-R_{\mathrm{infarct}}\right)\right)\right)\times \left(1.0+exp\left(-0.075\left(r_{2}-R_{\mathrm{infarct}}\right)\right)\right)}\\ +\frac{D_{\mathrm{GRF}}\left(x,y\right)}{\left(1.0+exp\left(-0.075\left(r_{1}-R_{\mathrm{border}}\right)\right)\right)\times \left(1.0+exp\left(-0.075\left(r_{2}-R_{\mathrm{border}}\right)\right)\right)}, \end{array}$$ (3)

where $r_{1}$ and $r_{2}$ were the Euclidean distances from (x,y) to the centre of each of the two regions of scar, $R_{\mathrm{infarct}}$ the radius of the simulated infarct zone, set to 12.5mm , and $R_{\mathrm{border}}$ the radius of the simulated border zone, set to 30mm . This process produced a smoothly changing diffusion field throughout the tissue. The values of the field were then adjusted so that all D(x,y) having a value greater than $0.1\;mm^{2}/ms$ were set to this value, and any D(x,y) with a value less than zero were set to zero.

This process is illustrated in Figure 1, which shows the diffusion fields obtained for length scales of 1.25 and 10.0mm .

The GRF was sampled 20 times at each of the 4 length scales, giving a total of 80 structural models.

### 2.4 Functional models of coupling to fibrotic scar

Six different models were then used to represent the coupling between excitable tissue, border zone, and scar. Each model was based on the assumption that diffusion (tissue conductivity) within both border zone and scar is reduced. The first three models used different representations of the diffusion field and boundary between excitable and inexcitable tissue, and in the final three we introduced random elements. Each model is specified below.

• ***ThresholdD*** In this model, tissue was assumed to be either fully excitable with uniform diffusion or inexcitable and uncoupled. $D_{\mathrm{ThresholdD}}\left(x,y\right)$ was therefore set to either $0.1\;mm^{2}/ms$ for $D\left(x,y\right)>0.025\;mm^{2}/ms$ , or zero otherwise. Regions with $D_{\mathrm{ThresholdD}}\left(x,y\right)$ of zero were set to be inexcitable by fixing the $I_{\mathrm{ion}}$ term in Equation 1 to zero, with no-flux boundaries.
• ***SmoothD*** In this model, tissue was assumed to be either fully excitable with smoothly varying diffusion, or inexcitable and uncoupled. $D_{\mathrm{SmoothD}}\left(x,y\right)$ varied smoothly with D(x,y) between $0.1\;mm^{2}/ms$ and $0.025\;mm^{2}/ms$ . In regions with $D\left(x,y\right)<0.025\;mm^{2}/ms$ , $D_{\mathrm{SmoothD}}\left(x,y\right)$ was set to zero. Regions with $D_{\mathrm{SmoothD}}\left(x,y\right)$ of zero were set to be inexcitable with no-flux boundaries.
• ***ContinuousD*** This model was similar to *SmoothD*, except that regions of inexcitable tissue were assumed to be coupled. $D_{\mathrm{ContinuousD}}\left(x,y\right)$ varied smoothly with D(x,y) between $0.1\;mm^{2}/ms$ and $0\;mm^{2}/ms$ . Regions with $D_{\mathrm{ContinuousD}}\left(x,y\right)<0.025\;mm^{2}/ms$ were set to be inexcitable, but diffusion was retained within these regions and no boundary condition was imposed. These regions were designated to represent fibrotic scar. Experimental observations of fibroblasts and myofibroblasts indicate resting potentials of between −10 and −50mV (Chilton et al., 2005; Kohl and Gourdie, 2014), and simulation studies have shown that under some conditions the elevated resting potential compared to surrounding tissue can initiate propagating waves (Sridhar et al., 2017). Initial simulations in our model showed that assigning fixed resting potentials of either −26mVor−45mV , typical of myofibroblasts and fibroblasts respectively, resulted in spontaneous activation of these regions. For this reason we set the initial condition of resting potential of inexcitable regions with $D\left(x,y\right)<0.025\;mm^{2}/ms$ to −86.2mV , in line with the resting potential of surrounding tissue.

Next, we constructed three further models with random removal of grid points around the edges of the simulated infarct to represent diffuse fibrosis.

• ***ThresholdD-random*** This model was a modification of *ThresholdD*, with random removal of grid points to represent small regions of diffuse fibrosis. A similar approach has been described in other studies as percolation (Roney et al., 2016) or random alterations to the connectivity matrix (Alonso and Bär, 2013). At each point (x,y) , a random number between 0 and one was generated. If this number was greater than $20.0\times D_{\mathrm{ThresholdD}}\left(x,y\right)$ , then the grid point was removed by setting $D_{\mathrm{ThresholdD}}\left(x,y\right)$ to be zero. The probability of removal was therefore zero for $D_{\mathrm{ThresholdD}}\left(x,y\right)\geq 0.05\;mm^{2}/ms$ , increasing to 0.5 at the edge of the excitable region. Regions with $D_{\mathrm{ThresholdD}}\left(x,y\right)$ of zero, including the removed points, were set to be inexcitable with no-flux boundaries.
• ***SmoothD-random*** This model was a modification of *SmoothD*, with random removal of grid points to represent small regions of patchy fibrosis as for *ThresholdD-random.*
• ***ContinuousD-random*** In this model, random removal was undertaken in the same way as for *SmoothD-random.* However, points that were removed remained coupled but inexcitable as with the *ContinuousD* model.

**FIGURE 2:**
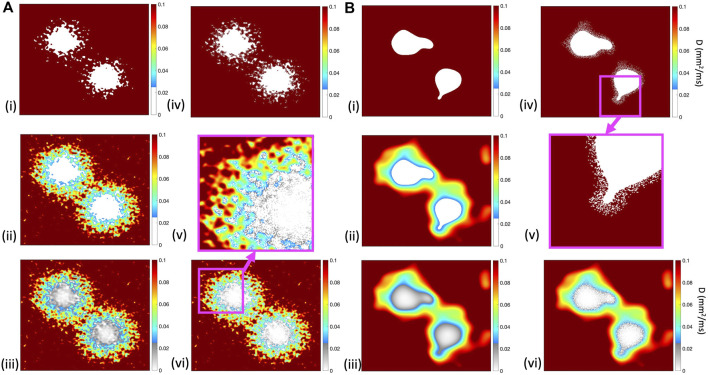
Diffusion fields for (i) *ThresholdD*, (ii) *SmoothD*, and (iii) *ContinuousD* models, with length scales of 1.25mm **(A)** and 10mm **(B)**, and colour bars in units of $mm^{2}/ms$ . In (i) and (ii), the inexcitable and uncoupled regions are shown as white, in (iii) the inexcitable and coupled regions are shown with diffusion represented as a greyscale. In (iv) and (vi), the diffusion fields for *ThresholdD-random* and *ContinuousD-random* are shown. Panels (v) show an enlargement of the diffusion field corresponding to the pink square in A(vi) and B(iv). Colour bars show diffusion coefficient, with grey regions denoting regions that are coupled but inexcitable.

Figure 2 illustrates the diffusion field for each model, showing long and short length scales.

### 2.5 Simulations and pacing protocol

Each tissue model was paced close to the isthmus by applying a stimulus current of −52pA/pF for 2ms to a circular region of radius 1.25mm in the lower left corner of the simulated tissue, 18.5mm from both the left hand and bottom edges. We delivered 3 S1 stimuli with a cycle length of 400ms , followed by an aggressive sequence of 5 S2 stimuli of the same strength and duration as S1, to expose possible re-entrant pathways. Our aim was to pace as rapidly as possible to simulate a rapidly activating focus, or stimuli from a re-entrant circuit and so during rapid pacing we delivered a stimulus as soon as the membrane voltage in the pacing region had fallen below −84.5mV . The coupling intervals of these rapid pacing stimuli were 323,232,255,227 , and 234ms (timings at 1111,1343,1598,1825 , and 2059ms ). These coupling intervals were consistent across the different models.

The duration of each simulation was 3.0 s. All 20×4× 6 = 480 simulations were run on local high performance computing (http://docs.hpc.shef.ac.uk), with each individual simulation run on a single core (2.4 kHz Intel Xeon) taking around 1.5 h to complete.

### 2.6 Post processing

The aim of post processing was to quantify the effect of simulated scar on activation and recovery during normal beats, and during the aggressive pacing protocol.

Activation and recovery times were determined at each location using a threshold crossing method with a threshold of −70mV , and these data were used to determine local activation time (LAT) and action potential duration (APD).

An additional simulation was run, with uniform tissue diffusion of $0.1\;mm^{2}ms^{-1}$ and no simulated scar. This simulation was used as a baseline to calculate differences in LAT (activation delay) and APD arising from both structural and functional models of scar.

Regions of tissue in which membrane voltage increased from beneath the threshold to above the threshold during a period of 20ms were denoted *active wavefronts*. An active wavefront represented the area traversed by a propagating action potential over 20ms , equivalent to isochrones of activation. Simulations retaining one or more active wavefronts at 3.0s were classified as supporting sustained re-entry. Simulations with one or more active wavefront at 2.6s but with no active wavefronts at 3.0s were classified as supporting transient re-entry.

## 3 Results

### 3.1 Effect of simulated scar on activation and APD

**FIGURE 3:**
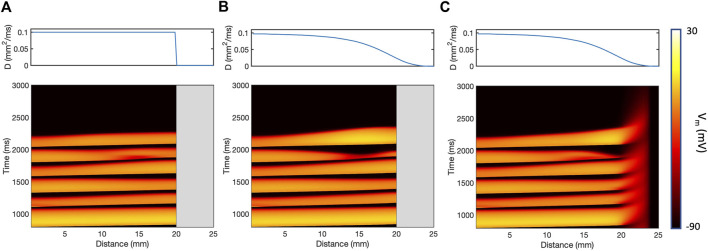
Action potential propagation in a thin 25×4mm strip of tissue for the *ThresholdD* **(A)**, *SmoothD* **(B)**, and *ContinuousD* **(C)** models. The top of each panel shows the variation of diffusion coefficient along the length of the strip, and the bottom shows a time and space plot of membrane voltage at the centre of the strip when paced at the left hand end. Greyed-out boxes indicate inexcitable and uncoupled regions of tissue.

The effect of the different models of fibrotic scar on action potential propagation in a 25×4mm strip of tissue with D varying smoothly along the tissue length is shown in Figure 3. These thin strips were paced using the same protocol as the larger tissue sheets, and each stimulus produced a propagating action potential. Figure 3 shows the final S1 beat, and the five premature beats. The short coupling intervals of the premature stimuli resulted in dynamic changes in APD and conduction velocity along the sheet. With the *SmoothD* and *ContinuousD* models, the change in D affected the fourth S2 beat, which was almost blocked in the *SmoothD* model, and was blocked in the *ContinuousD* model.

**FIGURE 4:**
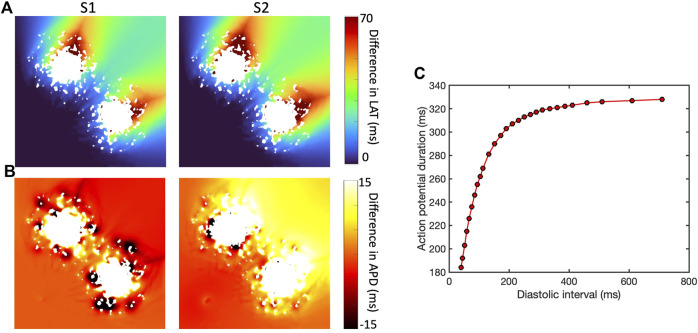
Effect of simulated scar on local activation time (LAT) **(A)** and APD **(B)**, for S1 beat at cycle length of Regional differences in APD for final S1 beat (cycle length 400ms ) and initial S2 beat (S1S2 coupling interval of 300ms ) for *ContinuousD* model with length scale of 1.25mm . In each case the colour indicates differences from a model with uniform diffusion. **(C)** Shows APD restitution curve in 2D tissue.

The presence of simulated fibrotic scar delayed activation and increased the dispersion of APD compared to a simulation without fibrotic scar. This behaviour is illustrated in Figure 4, which shows differences in LAT (Figure 4A) and APD (Figure 4B) observed in a representative sample of the *ContinuousD* model compared to observations in the model with uniform diffusion. LAT and APD differences are shown for an S1 beat (with cycle length of 400ms ) and for a premature S2 beat (with S1S2 coupling interval 300ms ). The simulated scar acted to delay activation for both S1 and S2 beats, consistent with local reductions in D .

In this example, the regions of short length scale scar acted to both increase and shorten APD for the S1 beat. The modest LAT delay associated with the premature S2 beat then acted to increase the diastolic interval beyond the scar, engaging APD restitution in the steep part of the curve (Figure 4C) and resulting in prolonged APD in this region.

**FIGURE 5:**
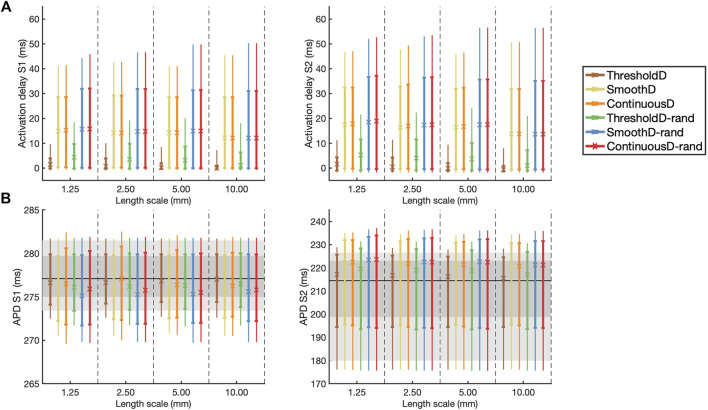
**(A)** Activation delay compared to simulation with uniform diffusion and **(B)** APD for the final S1 beat (left) and the initial S2 beat (right). The results are shown as median (symbol), inter-quartile range (thick line), and inter-decile range (thin line), where each measure is calculated across all 20 samples at each location. In **(B)** the horizontal black line indicates the median APD for simulation in uniform tissue; the dark and light grey regions show the inter-quartile and inter-decile range for this uniform simulation respectively.

These findings are extended and summarised across all models and simulations in Figure 5, which shows activation delay and APD for all four length scales and all six models of scar. Activation delay (Figure 5A) was calculated relative to the model with uniform diffusion $\left(D=0.1\;mm^{2}ms^{-1}\right)$ . Overall activation delay was smallest for the *ThresholdD* and *ThresholdD-random* models, which had a larger overall diffusion coefficient than the other models (see Figure 2). The spread of activation delays was a little greater for the S2 beat compared with the S1 beat, but there were no clear effects of length scale. In the *ThresholdD* and *ThresholdD-random* models the diffusion coefficient was set to $0.1\;mm^{2}/ms$ everywhere except for scar regions, and so the activation delay for these models shows that the effect of scar structure alone on activation was small, whereas spatially varying diffusion and coupling of the scar regions resulted in much more slowing of the activation wave.

Following the S1 beat, in all models median APD was slightly lower compared to uniform tissue, whereas following the S2 beat the median APD was slightly higher compared to uniform tissue (Figure 5B). In all models the inter-quartile and inter-decile ranges of APD, which are measures of APD dispersion, were greater than in uniform tissue. There were no clear effects of either length scale or fibrosis model on APD dispersion.

### 3.2 Vulnerability to re-entry

**FIGURE 6:**
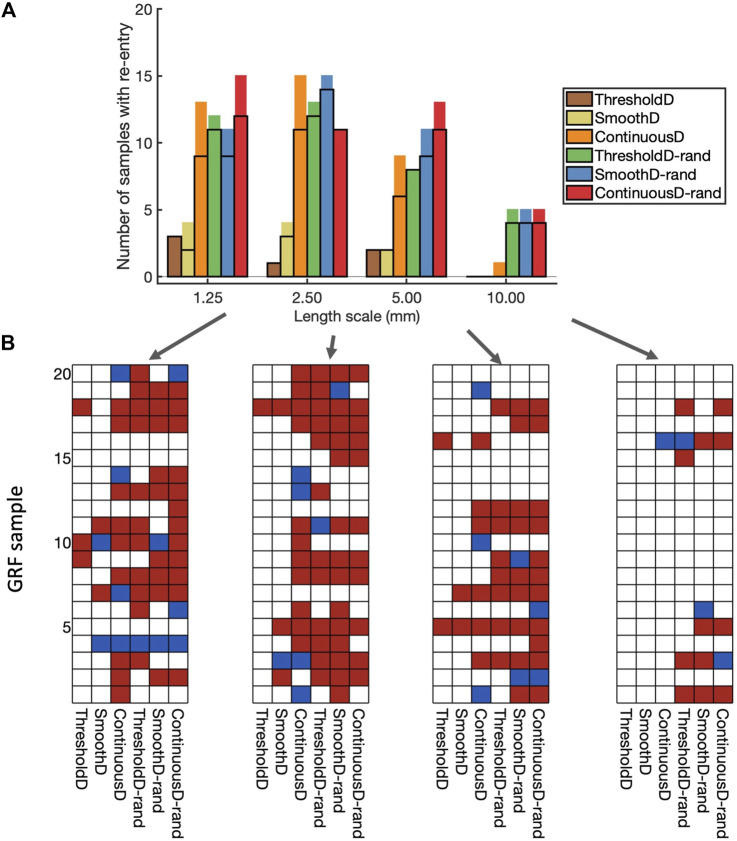
Incidence of re-entry in each model. **(A)** Overall view. Each bar shows the number of samples in which re-entry was produced by rapid pacing in each model. The part of the bar enclosed by black lines indicates the number of samples in which sustained re-entry was present at the end of the simulation (3.0 s), and the other part of the bar shows the number of samples in which transient re-entry was present at 2.6 s, but not at 3.0 s. **(B)** Grids showing the outcome of simulations with each of the 20 samples, and each of the nine fibrosis models. White indicates no re-entry, blue transient re-entry, and red sustained re-entry.

The incidence of re-entry in individual GRF samples depended on the both the length scale of simulated fibrosis and the scar model. Figure 6A shows the incidence of re-entry in each of the different models following aggressive pacing. In some simulations, no re-entry was elicited, in others re-entry terminated before the end of the 3.0s simulation period. We therefore distinguished between no re-entry, transient re-entry (active wavefronts at 2.6s but not 3.0s ), and sustained re-entry (active wavefronts at 3.0s ). Sustained re-entry occurred more often than transient re-entry, and the overall incidence of re-entry decreased as length scale increased.

The type of scar model had an important effect on the incidence of re-entry. With the *ThresholdD* and *SmoothD* models the incidence of sustained re-entry was low (≤3/20) , whereas for the *ContinuousD* model the incidence was relatively high (≥96/20) for length scales between 1.25 and 5mm .

Introducing randomness into the regions close to the simulated scar resulted in an increased incidence of both transient and sustained re-entry for the *ThresholdD-random* and *SmoothD-random* models. For the *ContinuousD-random* model, there was an increase in the incidence of sustained and transient re-entry at all length scales. The randomness used in our simulations is equivalent to small obstacles at the spatial resolution of the grid, which was 0.25mm . Thus the increased incidence of re-entry in models with randomness is consistent with the idea that obstacles with a smaller length scale act to initiate re-entry more effectively.

Figure 6B shows a different view of these results, where the incidence of re-entry is broken down by model and GRF sample. A total of 61 of the 80 GRF samples produced either transient or sustained re-entry with at least one of the fibrosis models. Some GRF samples favoured re-entry in most of the models, for example, sample 5 with length scale 5.0mm , whereas others, for example, sample 20 with length scale 5.0mm , were less favourable to re-entry.

These overall findings show that important roles in initiation of simulated re-entry are played by both the length scale and the configuration of scar and border zone regions (*i.e.*, the GRF sample). However, an even more important role is played by the way in which scar is represented in the model.

**FIGURE 7:**
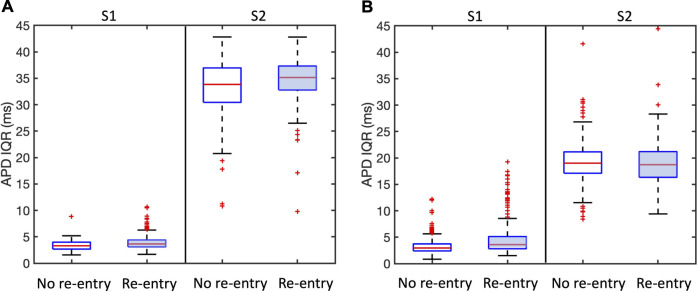
Association between APD dispersion measured by inter-quartile range (IQR) and incidence of sustained and transient re-entry. APD IQR was determined from the final S1 beat or the first S2 beat as shown. Plots are shown for all models. **(A)** shows results for the entire tissue sheet, and **(B)** results for the simulated infarct region alone. Each plot shows the median (red line), inter-quartile range (blue box), estimates of the extremes of the distribution (black lines), and outliers (red points).

APD dispersion has long been recognised as a measure of vulnerability to re-entry (Han and Moe, 1964), and in Figure 7 we show the overall APD IQR for simulations that resulted in sustained and transient re-entry, and those that did not. Figure 7A shows results for all simulations, and Figure 7B shows results for the region of scar, within 25mm of the centre of each lobe of the simulated infarct only. For S1 pacing, there is a weak indication that simulations with sustained re-entry may show a larger APD IQR during S1 pacing than those that do not. However, for S2 pacing there appears to be very little difference between the two groups.

We propose that the explanation for this observation is that APD IQR is an overall measurement of APD dispersion either locally or across the entire tissue, whereas re-entry is initiated as a result of much more local effects that may be very difficult to identify.

### 3.3 Mechanism and initiation of re-entry

**FIGURE 8:**
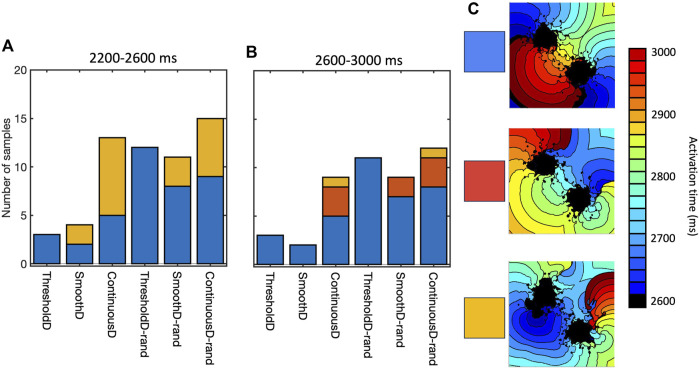
Mechanism of sustained re-entry in all six models with length scale of 1.25 mm. **(A,B)** number of samples with re-entry sustained by a figure-of-eight pattern with retrograde conduction through the isthmus (blue), re-entry around one lobe of the scar (gold), or a more complex re-entrant pattern (red), between 2,200 and 2,600 ms **(A)** and 2,600–3,000 ms **(B)**. **(C)** Local activation time plots showing examples of each re-entry mechanism.

Re-entry was often but not always sustained by retrograde activation through the isthmus between the main regions of scar, consistent with experimental and clinical evidence that highlights the importance of the isthmus (Anter et al., 2016; Pashakhanloo et al., 2018). However, we also observed re-entry around on lobe of the scar, as well as re-entry around small scale features distant from the scar. Figure 8 shows the mechanism of sustained re-entry for each of the different models at the shortest length scale. These mechanisms were determined from activation maps; examples are shown to illustrate each of the different mechanisms and all of the activation maps are included as Supplementary Material.

The prevalence of re-entry in models with the same structural representation of fibrotic scar (Figure 6), and the different mechanisms sustaining re-entry in the models of fibrosis (Figure 8) both indicate that the model of coupling between scar and normal tissue plays an important role in both initiating and sustaining re-entry.

**FIGURE 9:**
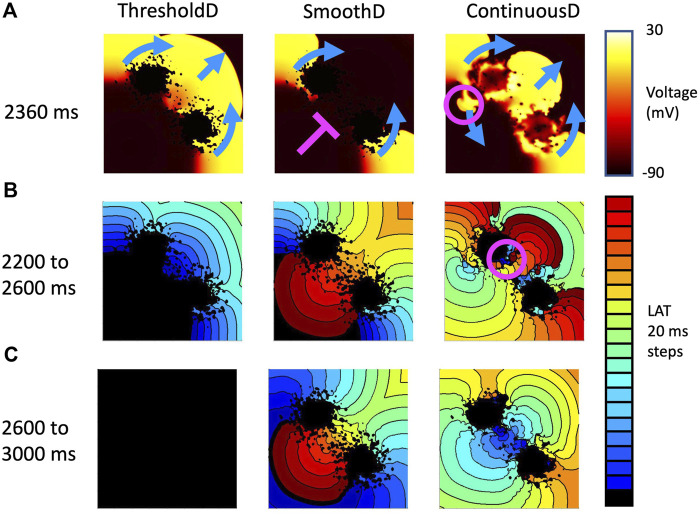
Example activation patterns in three fibrosis models for sample seven of tissue with a length scale of 1.25 mm. **(A)** Snapshot of membrane voltage in each model 2,360 ms after the start of the simulation. Propagation direction of the final premature beat (stimulus at 2059 ms) is shown by blue arrows. Pink line show where activation is blocked, and pink circle shows location of retrograde re-entry. **(B)** LAT between 2,200 and 2,600 s **(C)** LAT between 2,600 and 3,000 s.

This effect is illustrated in Figures 9A–C, which shows activation patterns in a single GRF sample where there was no re-entry, sustained re-entry, and transient re-entry in the *ThresholdD*, *SmoothD*, and *ContinuousD* models respectively. In the *ThresholdD* model, activation resulting from the final stimulus propagated around the two lobes of scar and through the isthmus as shown by the blue arrows. The activation wavefronts did not encounter recovering tissue and so there was no block and no subsequent re-entry. In the *SmoothD* model the activation wave propagated more slowly because the overall diffusion coefficient was lower. Propagation through the isthmus was initially blocked by recovering tissue, initiating figure-of-eight re-entry with retrograde propagation through the isthmus.

Activation patterns for the *ContinuousD* model were more complex. The activation wave resulting from the final stimulus propagated around the lobes of the scar and through the isthmus. However, delayed recovery in a small feature led to retrograde re-entry shown by the pink circle. This produced antegrade activation through the isthmus, which was followed by a further re-entrant breakthrough in the isthmus indicated by the pink circle in the right hand panel of Figure 9B. The resulting activation waves collided and extinguished re-entry.

### 3.4 Effect of random removal

**FIGURE 10:**
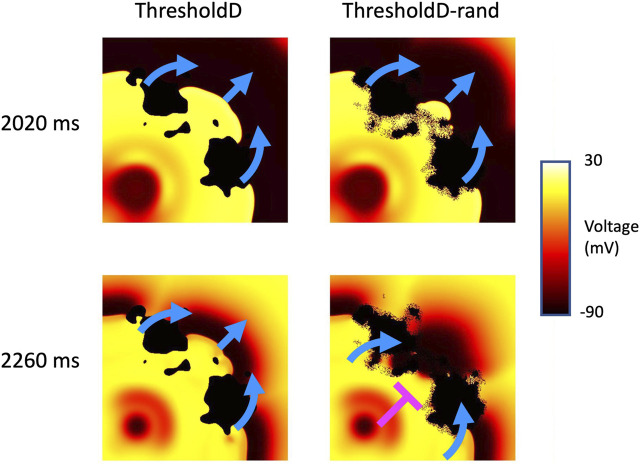
Block and re-entry in the *ThresholdD* model for sample 18 of tissue with a length scale of 10 mm. Snapshots of membrane voltage at 2020 and 2,260 ms show the penultimate and final beats of the stimulus sequence. Blue arrows show propagation, pink T shows block. See main text for details.

Random removal increased the incidence of re-entry, typically by block in the isthmus followed by retrograde activation through the isthmus (see Supplementary Material). An example is shown in Figure 10, which shows the behaviour of the *ThresholdD* model with and without random removal. In the *ThresholdD-rand* model the penultimate beat of the stimulus sequence propagated more slowly though the isthmus compared to the *ThresholdD* model. The final beat was then blocked by recovering isthmus tissue in the *ThresholdD-rand* model, leading to figure-of-eight re-entry.

## 4 Discussion

The main finding of this study is that in models of cardiac tissue where fibrotic scar is represented by spatially varying diffusion, the conditions under which re-entry is initiated by rapid stimulation are marginal, and are strongly influenced by the spatial scale of fibrotic features as well as the coupling between excitable and inexcitable tissue. The introduction of simulated fibrotic scar acted to delay activation and to increase dispersion of APD, but the magnitude of these changes was not strongly affected by either the fibrosis model or the spatial scale of fibrosis.

The detailed electrophysiology of scar and border zone remains poorly characterised, and so we do not propose that any of these models is better than another. An important next step is to work in tandem with experiments to determine the most appropriate model, and then to undertake further work to better understand the mechanisms that promote block and re-entry. Nevertheless, we have shown that the choice of model is important and should borne in mind for simulations where scar or infarct are to be included. Fibrotic scar is a potential therapeutic target (Simon-Chica et al., 2023), and so a deeper understanding of the mechanisms underlying scar electrophysiology has important practical significance.

### 4.1 Representation of fibrotic scar

We used Gaussian random fields to generate diffusion fields that vary smoothly in space and have a well defined length scale. This approach enables a set of samples to be drawn with the same overall characteristics and overall texture, but with a different pattern in each sample so that a range of scar configurations can be explored. Use of GRFs has allowed us to explore the range of possible tissue/fibrosis pathways that could generate re-entry. We stress that the purpose of this study was not to find an optimum representation of fibrotic scar in computational models, but rather to highlight the fact that re-entry involving these complex structures is a marginal event that depends on many factors including how excitable and inexcitable tissue, as well as their coupling, is represented numerically. Other approaches for generating tissue texture could also be used, including Perlin noise (Jakes et al., 2019), and careful validation of these and other models against experimental images would be a valuable next step for generating representative and realistic patterns of fibrotic scar.

Our use of changes in diffusion (tissue conductivity) alone to represent the effect of fibrotic scar is based on evidence from both tissue culture (Gaudesius et al., 2003) and experimental preparations (Ghouri et al., 2018) showing that fibrotic scar acts to slow action potential propagation. However, this is a limitation because scar is a complex mixture of myocytes, fibroblasts, collagen, and other non-myocytes (Rog-Zielinska et al., 2016), and diffusion may be a rather blunt instrument to represent these. Although these structural features alone have been found to be sufficient to produce re-entry in computational models (Engelman et al., 2010), other simulations have shown that a more detailed representation of non-myocytes enables more insight into the way that action potentials can propagate through fibrotic scar (Simon-Chica et al., 2023). Explicit representation of the fibroblast space (Sachse et al., 2009), as well as recent work on homogenisation of these complex structures (Farquhar et al., 2022; Lawson et al., 2023) and myocyte-fibroblast interactions (Sridhar and Clayton, 2024) are further promising developments that will enable more representative models to be developed.

Consistent with an earlier study in simulated atrial tissue (Clayton, 2018), we found that fibrosis with a shorter length scale was in general more likely to produce and sustain re-entry. Including even smaller scale heterogeneity by random removal of grid points also tended to increase the incidence of re-entry. However, the magnitude of this effect was small relative to the differences between the different models, suggesting that the way that normal myocardium and scar is coupled plays an important role.

### 4.2 Limitations

Faithful representation of discrete and heterogeneous tissue in numerical and computational models is challenging because the standard models of cardiac electrophysiology assume that excitable tissue can be treated as a functional syncytium (Clayton et al., 2011). It is important to consider carefully how spatial variation in tissue conductivity and coupling in the transition zone between excitable and inexcitable regions is handled. In the present study, we have represented this transition in different ways, and have shown that different boundary conditions and coupling influence the incidence and persistence of re-entry. The reason for this finding appears to be the marginal conditions for propagation and block in regions of tissue with decreased conductivity as illustrated in Figures 9, 10. Our observations are consistent with the ideas that small variations in diffusive current flow in the different models act to tip the balance between propagation and block.

An important limitation of our study is that we have only examined behaviours in 2D tissue sheets, and the reason for this choice was the lower computational demands of 2D simulations, and the ease of interpreting the simulation outputs. Further work in 3D and whole-ventricle models incorporating anisotropic diffusion are an important next step, which will enable the way that anisotropic diffusive current flow affects the marginal conditions around propagation and block. More detailed and personalised models of scar based on clinical data are a longer term objective (Lopez-Perez et al., 2019), which will allow the effect of different ablation strategies to be explored.

The model of scar and border zone used in this study was based on superposition of spatially varying fields describing the infarct and border zone (Eqs 2, 3), with these fields being defined by a logistic function of the distance from the centre of the simulated infarct. We chose this approach because it resulted in a smooth weighting of $D_{\mathrm{GRF}}$ within the border zone, and for simplicity we chose to use two identical logistic functions for infarct and border zone. A valuable extension of the present study would be to investigate in detail how the properties and dimension of the border zone affects propagation and block.

Furthermore, the potency of simulated fibrotic scar substrates almost certainly depend on the model of cellular electrophysiology that is used as well as the dimensions of the scar and the pacing protocol. In this study we used the TNNP06 model with a single parameter set (ten Tusscher and Panfilov, 2006) and a fixed scar size. We would expect that different parameter sets, spatial heterogeneity in parameters, different cellular electrophysiology models, different scar geometry, and different pacing protocols would all affect the inducibility and persistence of simulated re-entry. However, the purpose of this study was to investigate different representations of fibrotic scar, and so we used a single cellular electrophysiology model and parameter set to provide a consistent baseline. Nevertheless, future detailed mechanistic studies of re-entry in the presence of fibrotic scar, ideally conducted in tandem with experiments, will need to ensure that model dependent effects are carefully taken into account.

The six different models of scar used in this study embraced a range of different plausible representations. Other approaches have also been described (Connolly and Bishop, 2016). Without detailed comparison to experimental and structural data, it is not possible to judge which of the models examined in the present study is most representative of fibrotic scar in real hearts.

### 4.3 Basic science and potential clinical significance

In the present study we typically observed re-entry with retrograde conduction through isthmus resulting most of the time in figure-of-eight ventricular tachycardia as documented in experiments (Ciaccio et al., 2022). This was an expected finding because we chose to represent two regions of fibrotic scar with a central isthmus. Few simulations resulted in multiple wavefronts and VF despite our choice of a parameter set for the TNNP06 model that results in steep APD restitution and breakup of spiral waves (ten Tusscher and Panfilov, 2006). It is possible that breakup of the initial re-entry might have been seen in longer simulations, but another explanation may be slow conduction through the isthmus. The typical cycle of re-entry through the isthmus was 200−250ms , and so the steep part of the APD restitution curve was not accessed by these re-entrant waves.

Our study highlights the possibly important role of microstructure in initiation of re-entry, and the potential value of therapeutic interventions targeted at these small scale features (Pashakhanloo et al., 2018). However, we have also found that it may be hard to identify these small scale features that act as substrates for re-entry. Figure 7 showed negligible differences in APD dispersion between samples that supported re-entry and those that did not, even when measures of APD dispersion were restricted to the neighbourhood of the infarct. More focused methods such as the re-entry vulnerability index, which is a measure of vulnerability to re-entry based on relative measurements of activation and recovery within a prescribed region (Campos et al., 2019) could provide a way to refine this approach.

Recent work on using computational models to identify the re-entrant circuits that sustain ventricular tachycardia in real patients has shown great promise (Prakosa et al., 2018; Zhang et al., 2023). It is intriguing that the present study has highlighted that the marginal conditions for re-entry can depend on the details of the model implementation, whereas the studies in patients have strong predictive power. Further studies to assess the relative importance of detailed and patient specific structural and function representations of fibrotic scar could address this interesting question.

## Data Availability

The original contributions presented in the study are included in the article/Supplementary Material, further inquiries can be directed to the corresponding author. All code used for the simulations described in this paper is publicly available at https://github.com/RichardClayton/VentricularFibrosis.
