# Supplementary material for: Re-entry in models of cardiac ventricular tissue with scar represented as a Gaussian random field
Source: Front Physiol. 2024 Jun 28;15:1403545. doi: 10.3389/fphys.2024.1403545 (PMC11239552; doi:10.3389/fphys.2024.1403545)
Supplement: Supplementary file 1 [file DataSheet1.PDF]

Supplementary Material: Re-entry in models of cardiac ventricular tissue with scar represented as a Gaussian random field

Richard H Clayton[1] and S. Sridhar[1]

[1] *Insigneo Institute for in-silico Medicine and Department of Computer Science, University of Sheffield, Sheffield, S10 2TN, United Kingdom*

1 Introduction

We have included details of the algorithm used to generate Gaussian random fields, as well as a series of plots showing the active wavefronts in each model of fibrotic scar immediately following the final stimulus at 2059 ms.

2 GRF algorithm

We used the following algorithm for generating GRFs, with an implementation based on Matlab code.

- Define the size of the GRF, in our case a 2D sheet of size $N = 400$.
- Define the covariance function ρ and lengthscale λ. We used a squared exponential covariance function $\rho(i, j) = exp\left(-(i^2 + j^2)/\lambda^2\right)$.
- Assemble $N \times N$ matrix $\mathbf{Rows} = \rho(i, j)$ where $i = 1 \dots N$ and $j = 1 \dots N$..
- Create the first row of the block circulant matrix, rearranging to an $(2N - 1) \times (2N - 1)$ matrix $\mathbf{B}$ with four blocks:
  1. $\mathbf{B}(1 \dots N, 1 \dots N) = \mathbf{Rows}(1 \dots N, 1 \dots N)$,
  2. $\mathbf{B}(1 \dots N, N + 1 \dots 2N - 1) = \mathbf{Rows}(1 \dots N, N \dots 2)$,
  3. $\mathbf{B}(N + 1 \dots 2N - 1, 1 \dots N) = \mathbf{Rows}(N \dots 2, 1 \dots N)$,
  4. $\mathbf{B}(N + 1 \dots 2N - 1, N + 1 \dots 2N - 1) = \mathbf{Rows}(N \dots 2, N \dots 2)$.
- Compute eigenvalues of $\mathbf{B}$ rearranged as a $(2N - 1) \times (2N - 1)$ matrix using a scaled 2D FFT, $\mathbf{\Gamma} = Real\left(FFT2(\mathbf{B})\right)/(2N - 1)^2$.
- Providing $\mathbf{\Gamma}$ is positive definite, calculate the $(2N - 1) \times (2N - 1)$ matrix $\mathbf{F} = FFT2(\mathbf{\Gamma} \circ \mathbf{R})$, where $\mathbf{R}$ is a matrix of complex numbers with real and imaginary parts obtained from a Gaussian distribution with mean of zero and standard deviation 1.0, and $\circ$ denotes Hadamard (element-wise) multiplication.
- The real part of $\mathbf{F}(1 : N, 1 : N)$ is then an $N \times N$ GRF.

3 Active wavefront plots

For each length scale, the active wavefront plots have two panels. The first panel (a) shows active wavefronts in each of the 20 GRF samples from 2200 to 2580 ms, and the second panel (b) shows active wavefronts in each sample from 2600 to 2980 ms.

Each plot is coloured to show local activation time at 20 ms intervals, and colours have been assigned as indicated in the colour bar. Black indicates the start time for each panel (2200 or 2600 ms), and red shows the end time (2580 or 2980 ms). Panels that are completely black show now active wavefronts, and in these simulations activation had ceased.

3.1 *ThresholdD* model

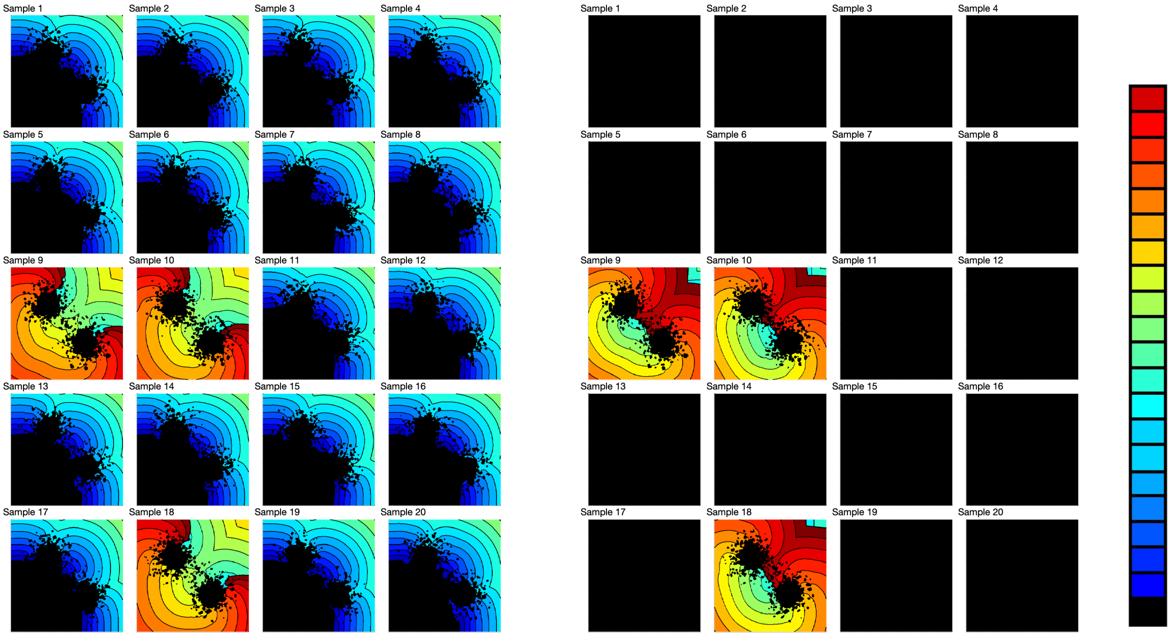

(a) 2200 ms to 2580 ms

(b) 2600 ms to 2980 ms

Figure 1: *ThresholdD* model, length scale 1.25 mm.

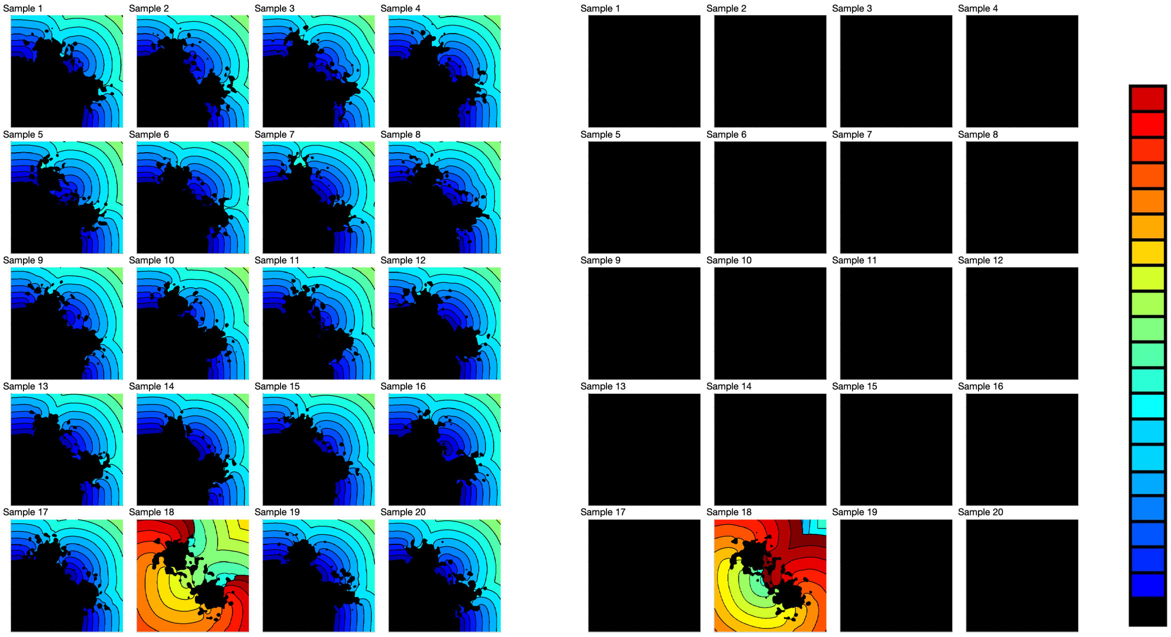

(a) 2200 ms to 2580 ms

(b) 2600 ms to 2980 ms

Figure 2: *ThresholdD* model, length scale 2.5 mm.

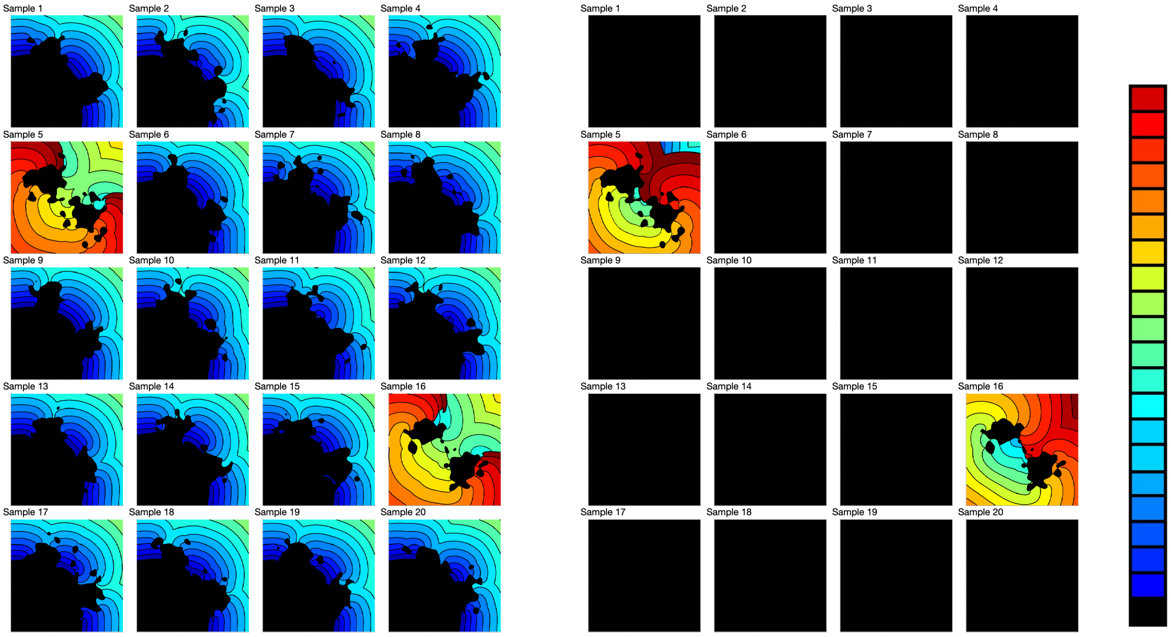

(a) 2200 ms to 2580 ms

(b) 2600 ms to 2980 ms

Figure 3: *ThresholdD* model, length scale 5.0 mm.

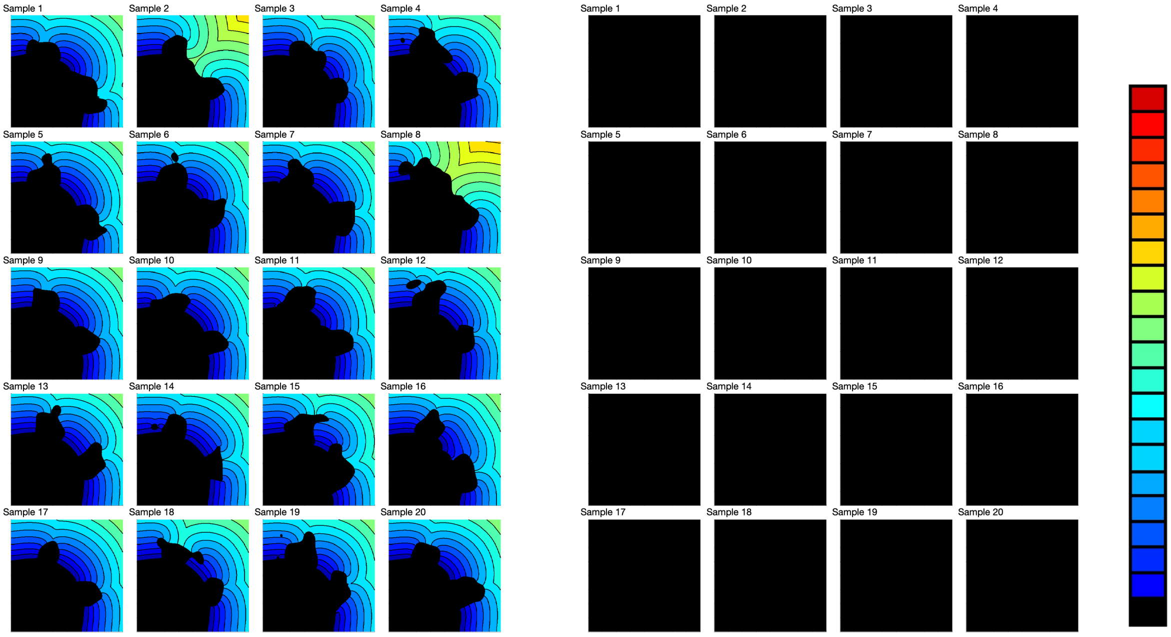

(a) 2200 ms to 2580 ms

(b) 2600 ms to 2980 ms

Figure 4: *ThresholdD* model, length scale 10.0 mm.

3.2 *SmoothD* model

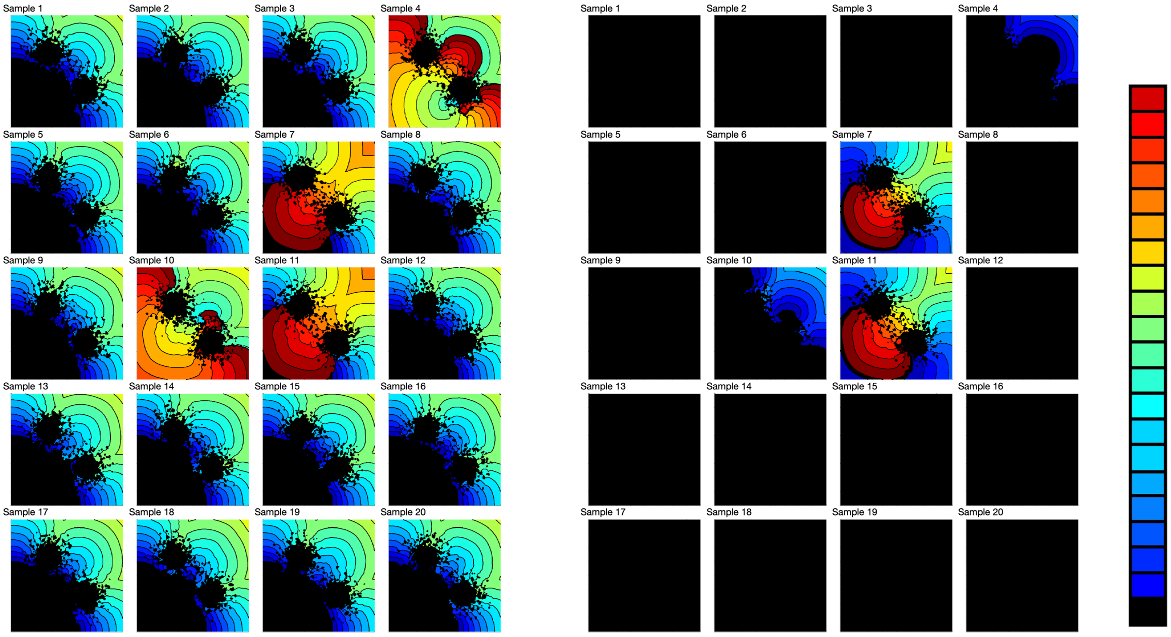

(a) 2200 ms to 2580 ms

(b) 2600 ms to 2980 ms

Figure 5: *SmoothD* model, length scale 1.25 mm.

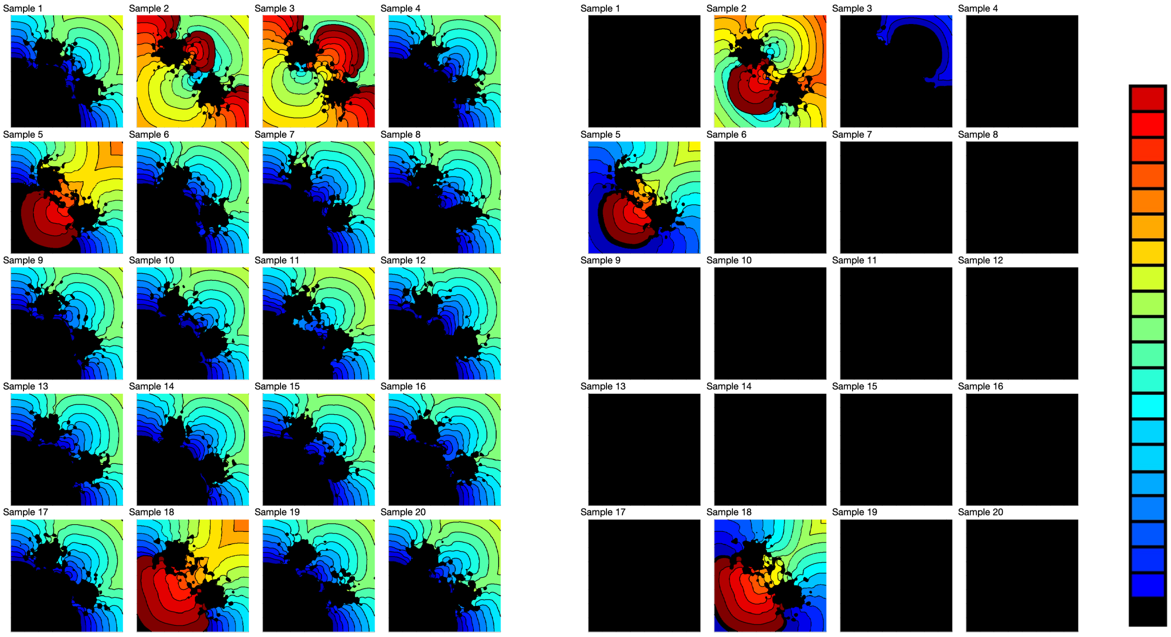

(a) 2200 ms to 2580 ms

(b) 2600 ms to 2980 ms

Figure 6: *SmoothD* model, length scale 2.5 mm.

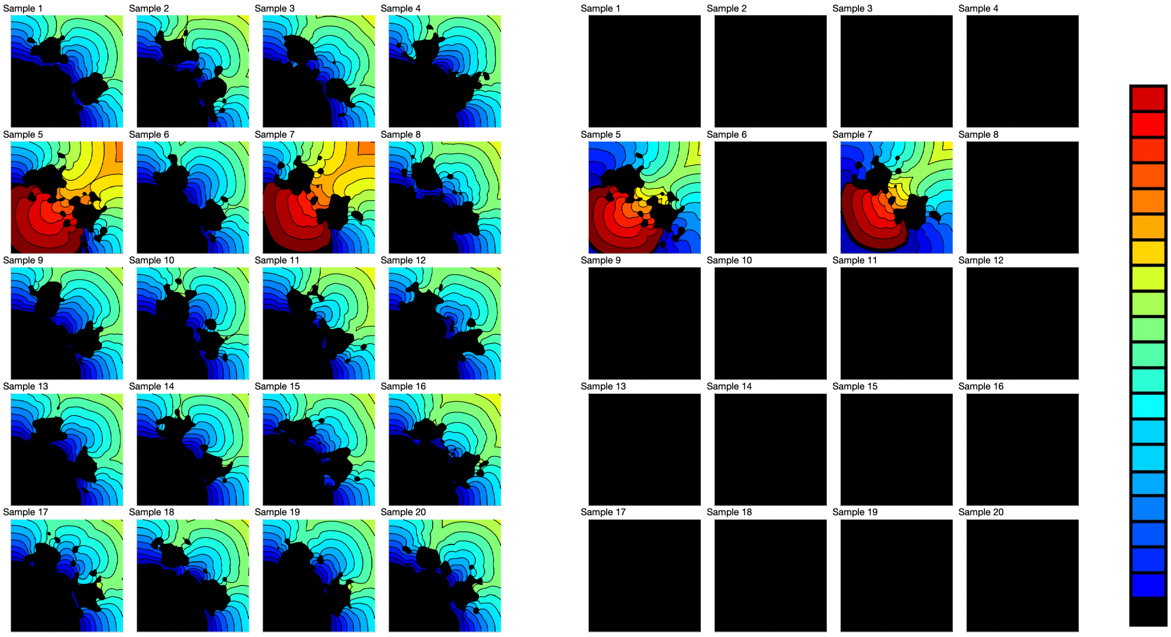

(a) 2200 ms to 2580 ms

(b) 2600 ms to 2980 ms

Figure 7: *SmoothD* model, length scale 5.0 mm.

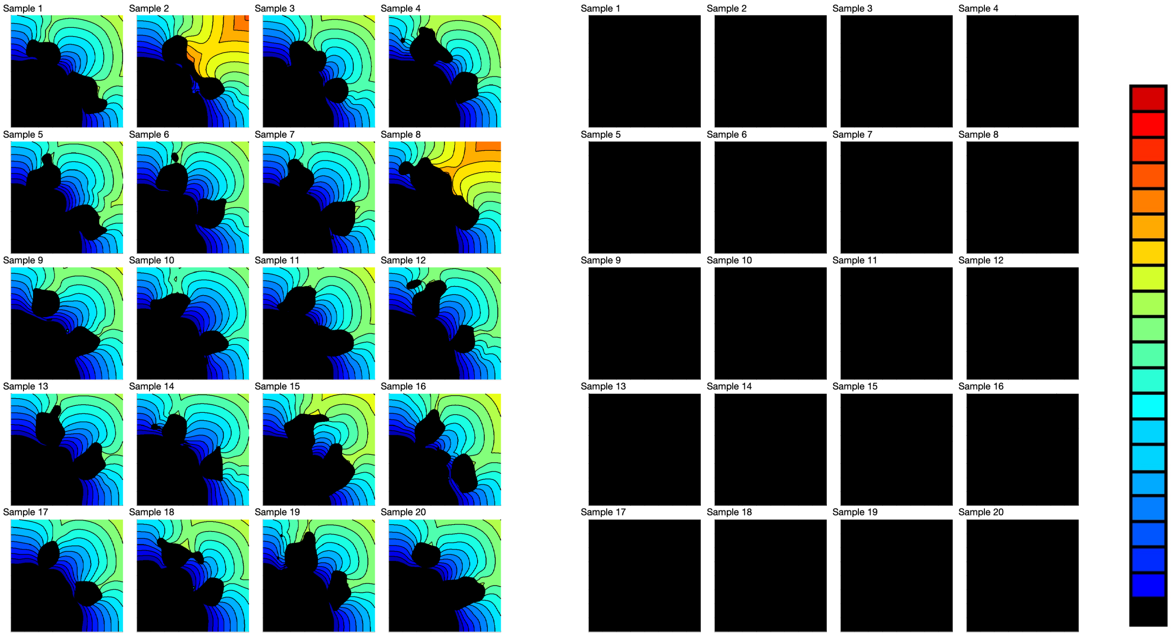

(a) 2200 ms to 2580 ms

(b) 2600 ms to 2980 ms

Figure 8: *SmoothD* model, length scale 10.0 mm.

3.3 *ContinuousD* model

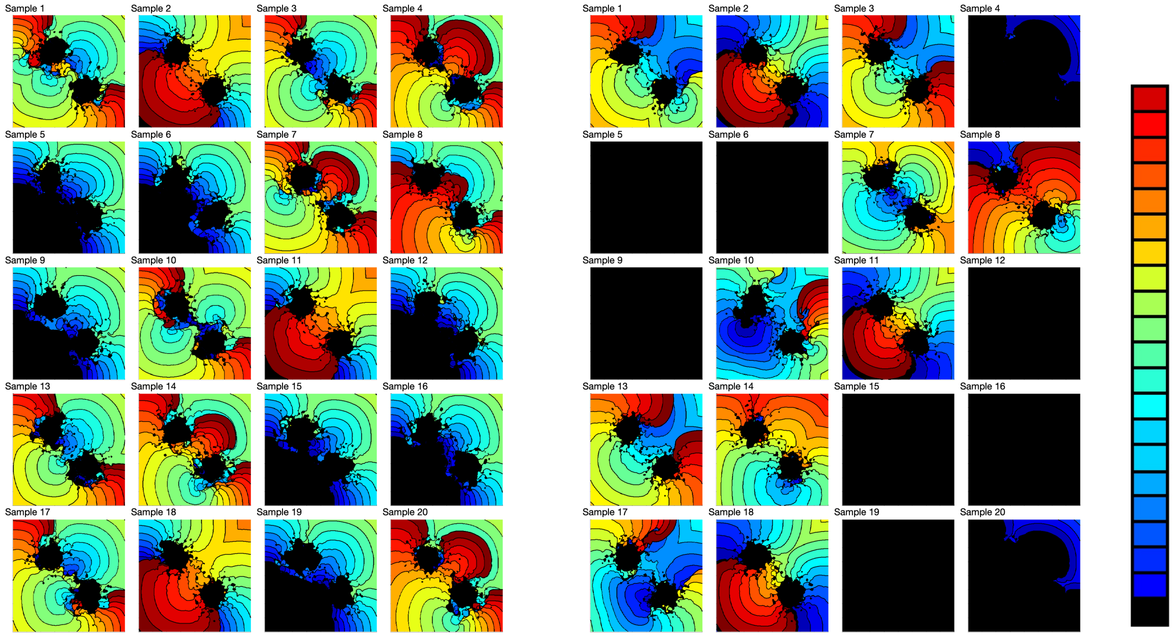

(a) 2200 ms to 2580 ms

(b) 2600 ms to 2980 ms

Figure 9: *ContinuousD* model, length scale 1.25 mm.

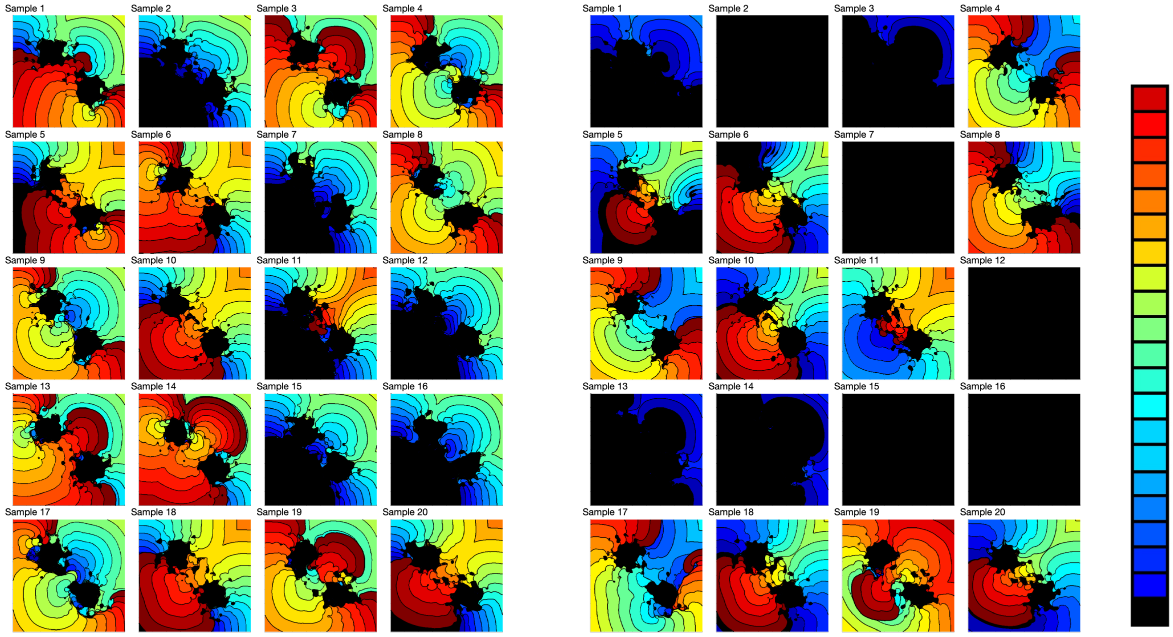

(a) 2200 ms to 2580 ms

(b) 2600 ms to 2980 ms

Figure 10: *ContinuousD* model, length scale 2.5 mm.

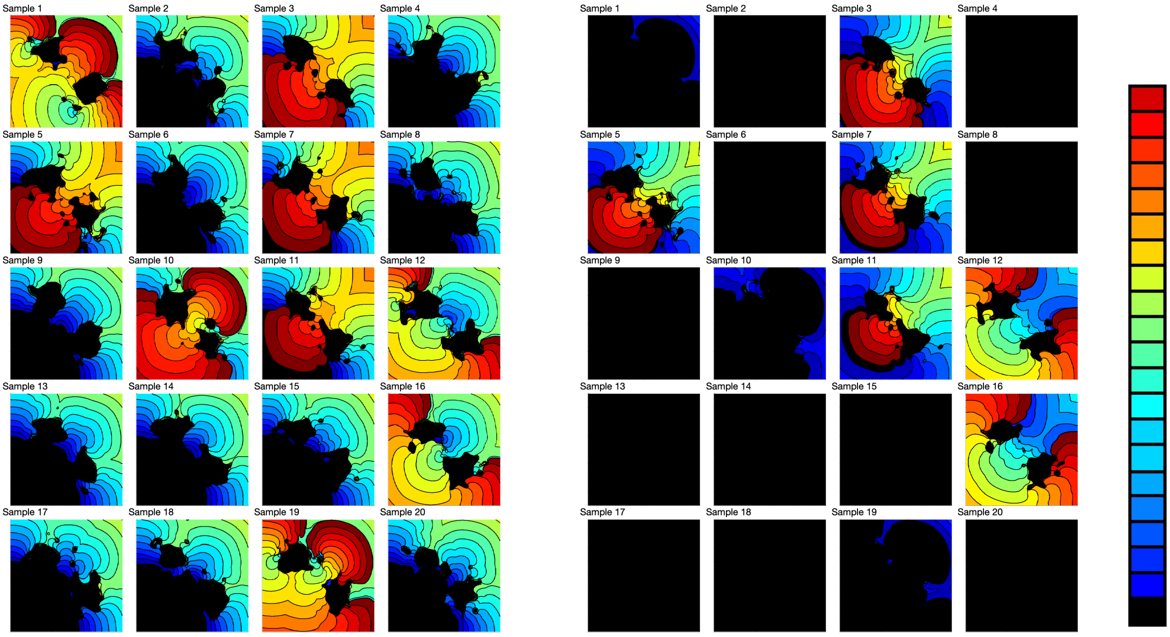

(a) 2200 ms to 2580 ms

(b) 2600 ms to 2980 ms

Figure 11: *ContinuousD* model, length scale 5.0 mm.

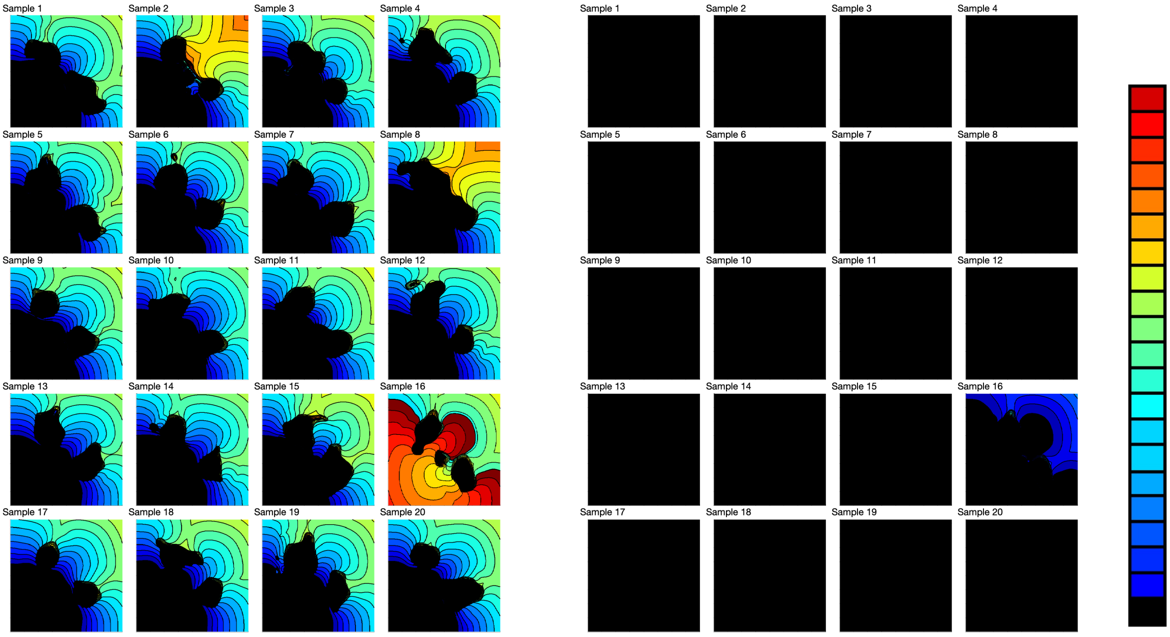

(a) 2200 ms to 2580 ms

(b) 2600 ms to 2980 ms

Figure 12: *ContinuousD* model, length scale 10.0 mm.

3.4 *ThresholdD-random* model

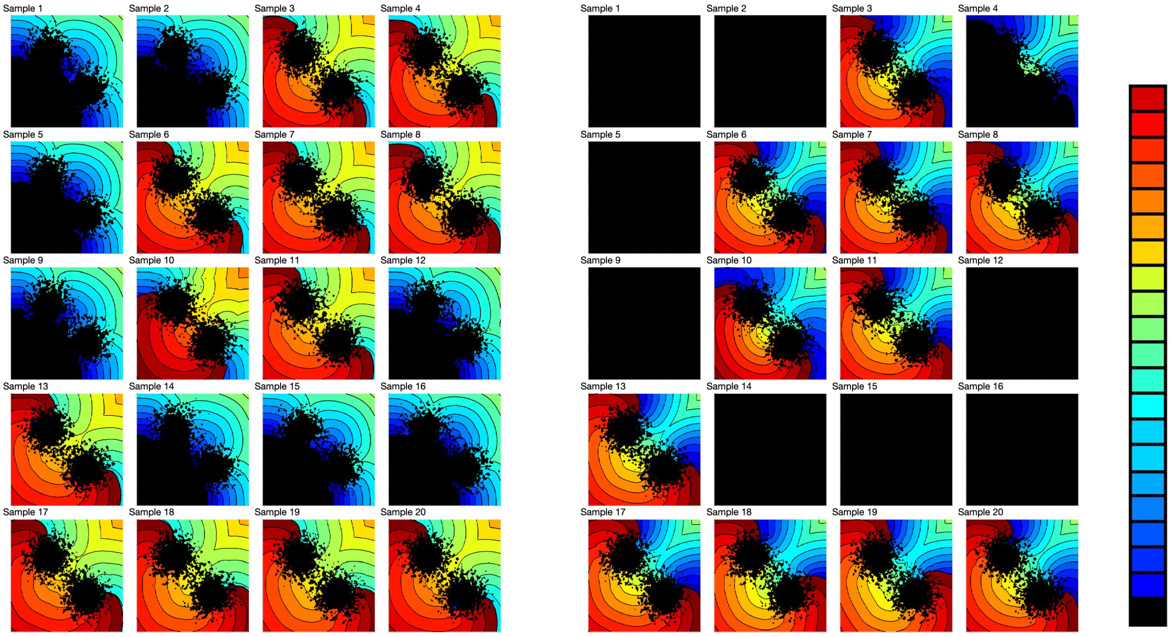

(a) 2200 ms to 2580 ms

(b) 2600 ms to 2980 ms

Figure 13: *ThresholdD-random* model, length scale 1.25 mm.

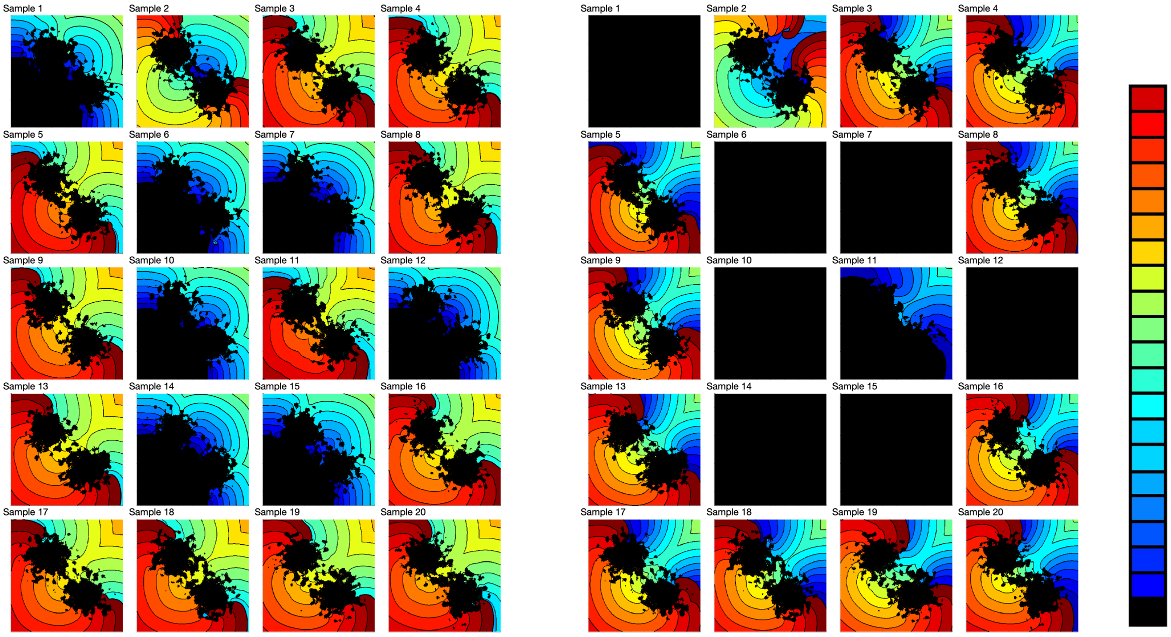

(a) 2200 ms to 2580 ms

(b) 2600 ms to 2980 ms

Figure 14: *ThresholdD-random* model, length scale 2.5 mm.

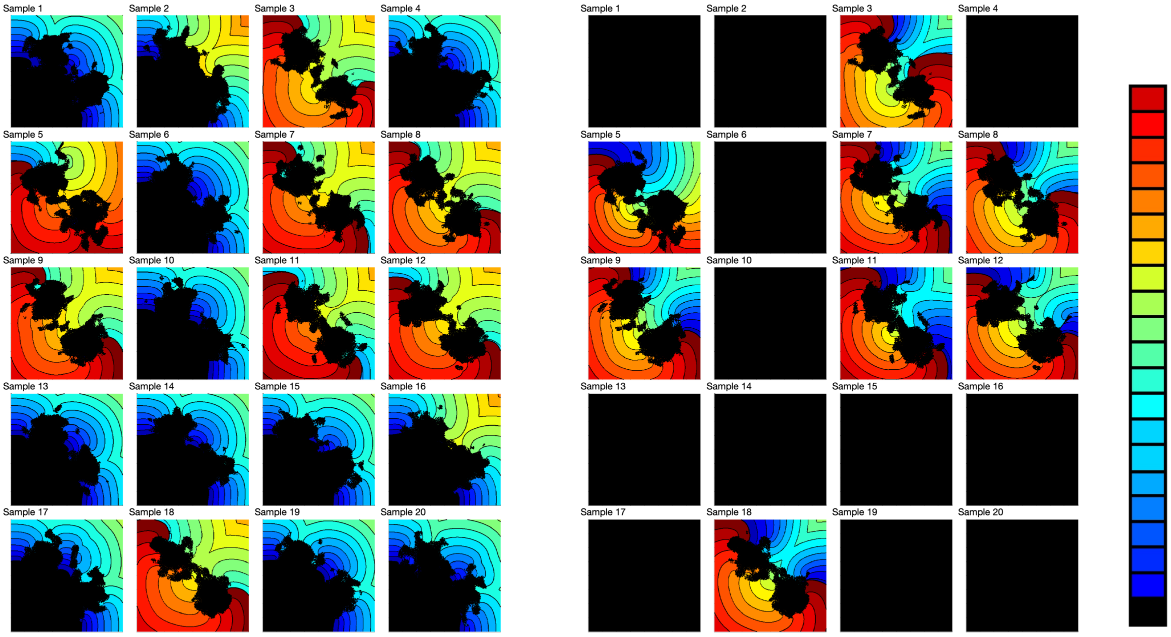

(a) 2200 ms to 2580 ms

(b) 2600 ms to 2980 ms

Figure 15: *ThresholdD-random* model, length scale 5.0 mm.

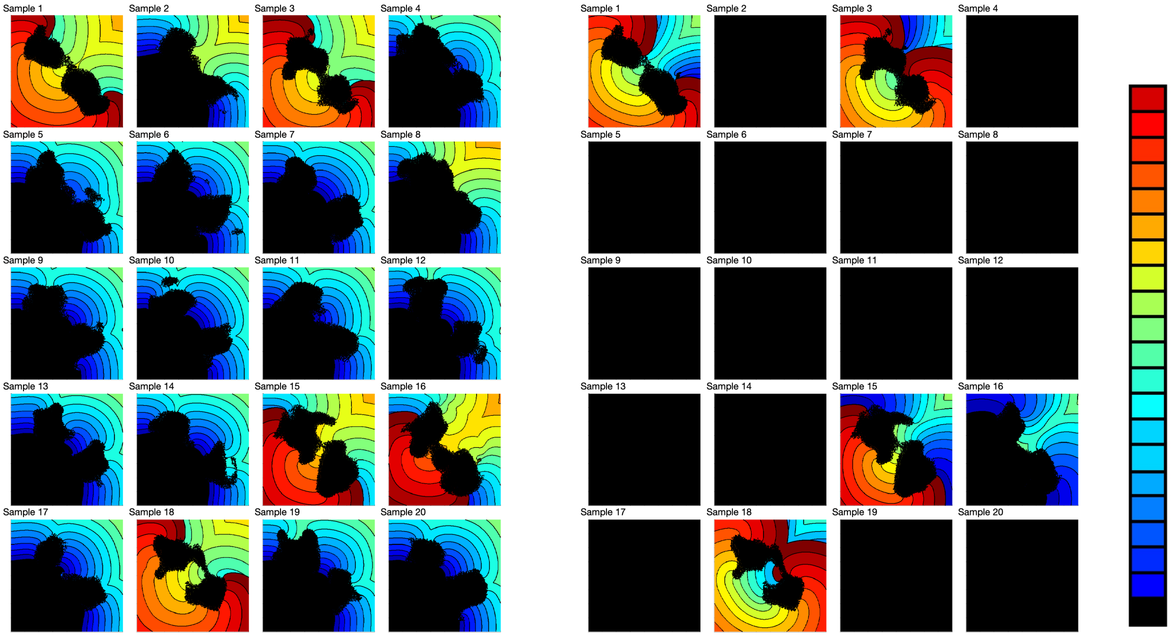

(a) 2200 ms to 2580 ms

(b) 2600 ms to 2980 ms

Figure 16: *ThresholdD-random* model, length scale 10.0 mm.

3.5 ***SmoothD-random*** model

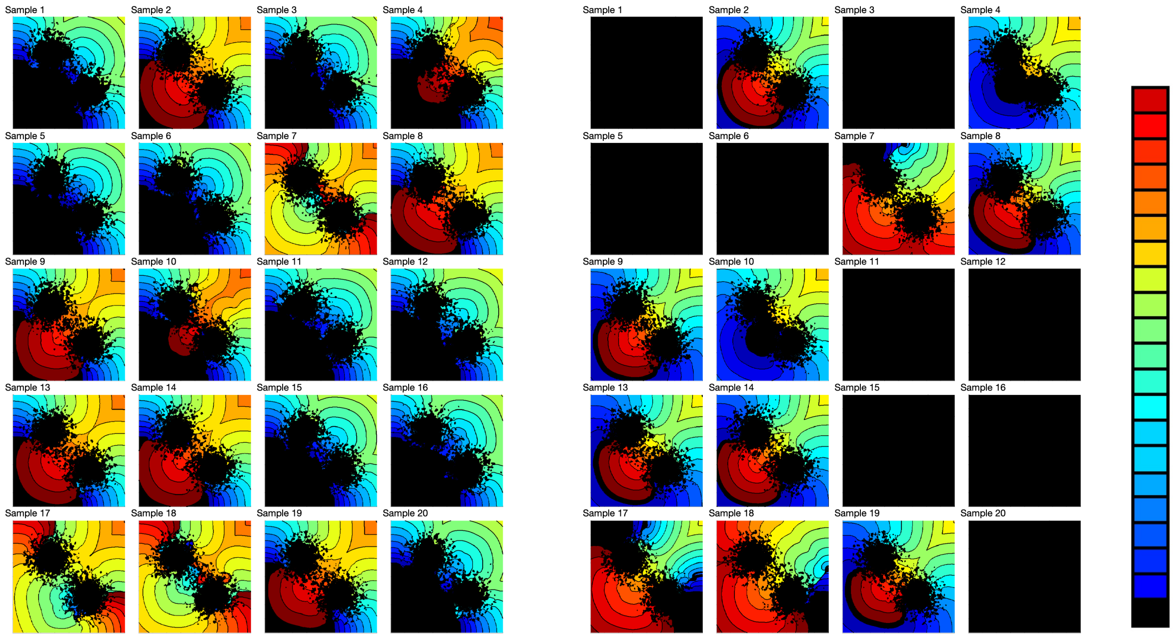

(a) 2200 ms to 2580 ms

(b) 2600 ms to 2980 ms

Figure 17: *SmoothD-random* model, length scale 1.25 mm.

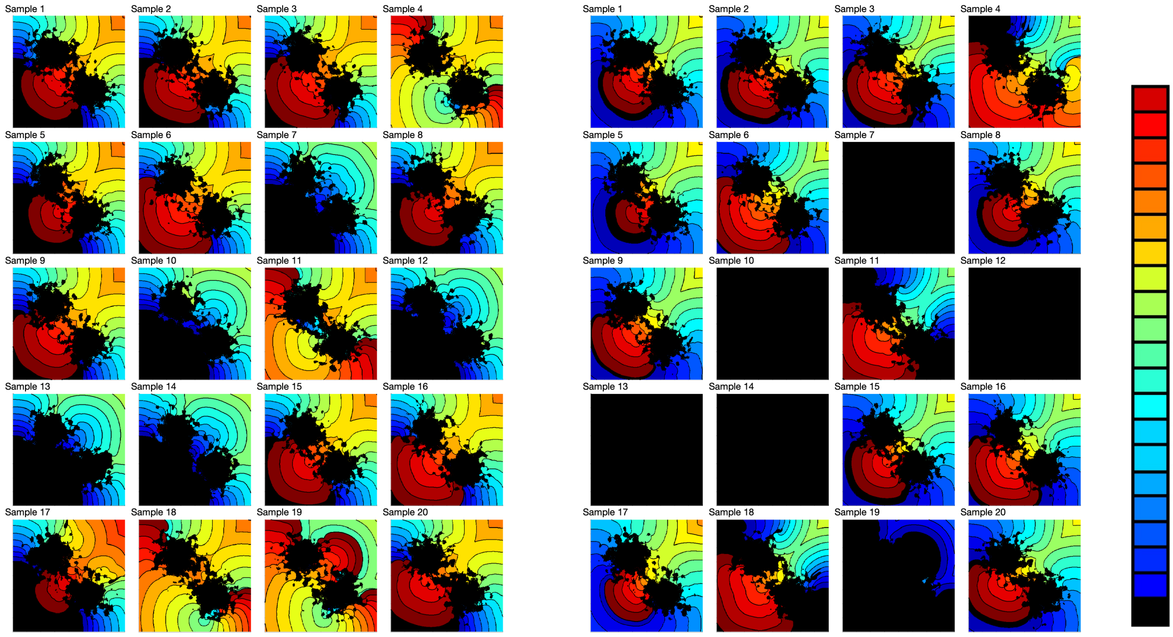

(a) 2200 ms to 2580 ms

(b) 2600 ms to 2980 ms

Figure 18: *SmoothD-random* model, length scale 2.5 mm.

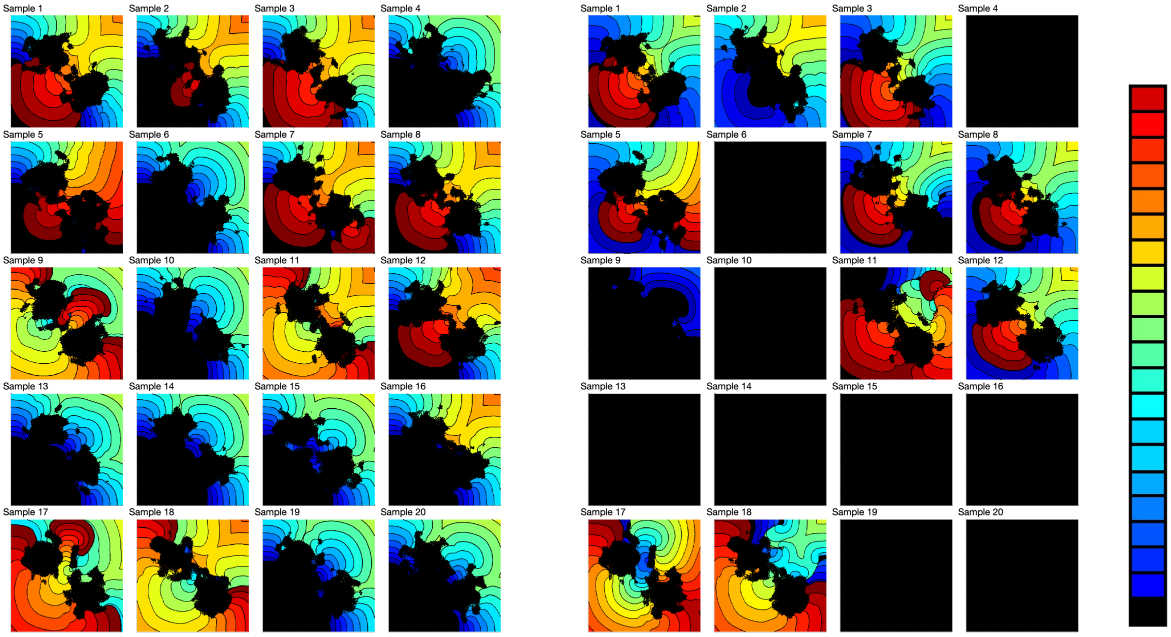

(a) 2200 ms to 2580 ms

(b) 2600 ms to 2980 ms

Figure 19: *SmoothD-random* model, length scale 5.0 mm.

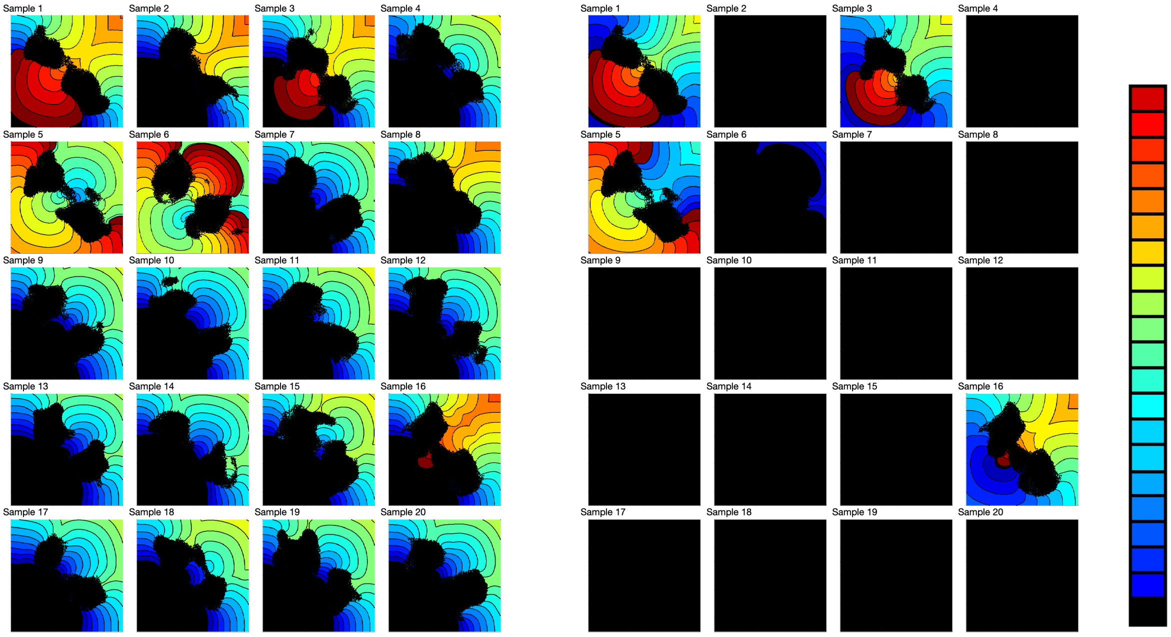

(a) 2200 ms to 2580 ms

(b) 2600 ms to 2980 ms

Figure 20: *SmoothD-random* model, length scale 10.0 mm.

3.6 *ContinuousD-random* model

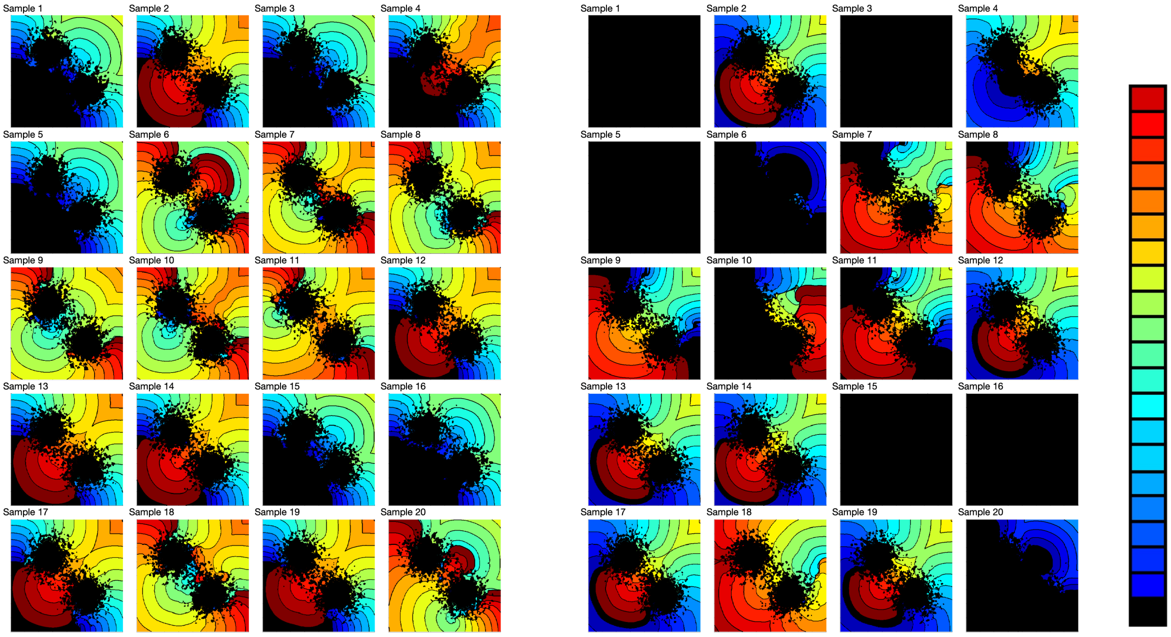

(a) 2200 ms to 2580 ms (b) 2600 ms to 2980 ms

Figure 21: *ContinuousD-random* model, length scale 1.25 mm.

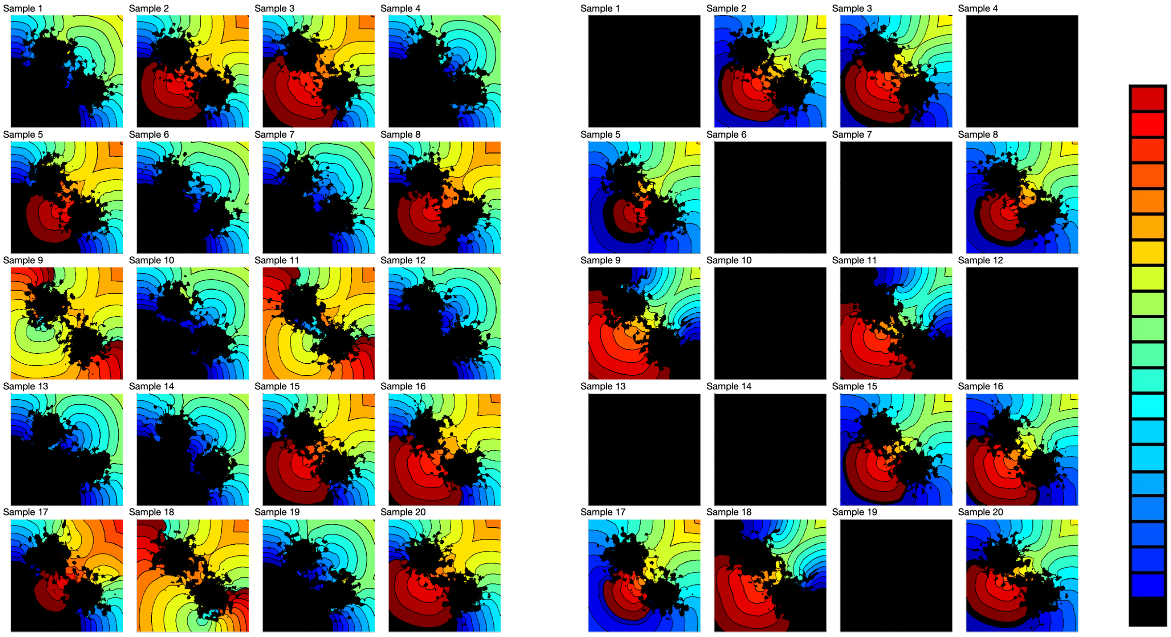

(a) 2200 ms to 2580 ms (b) 2600 ms to 2980 ms

Figure 22: *ContinuousD-random* model, length scale 2.5 mm.

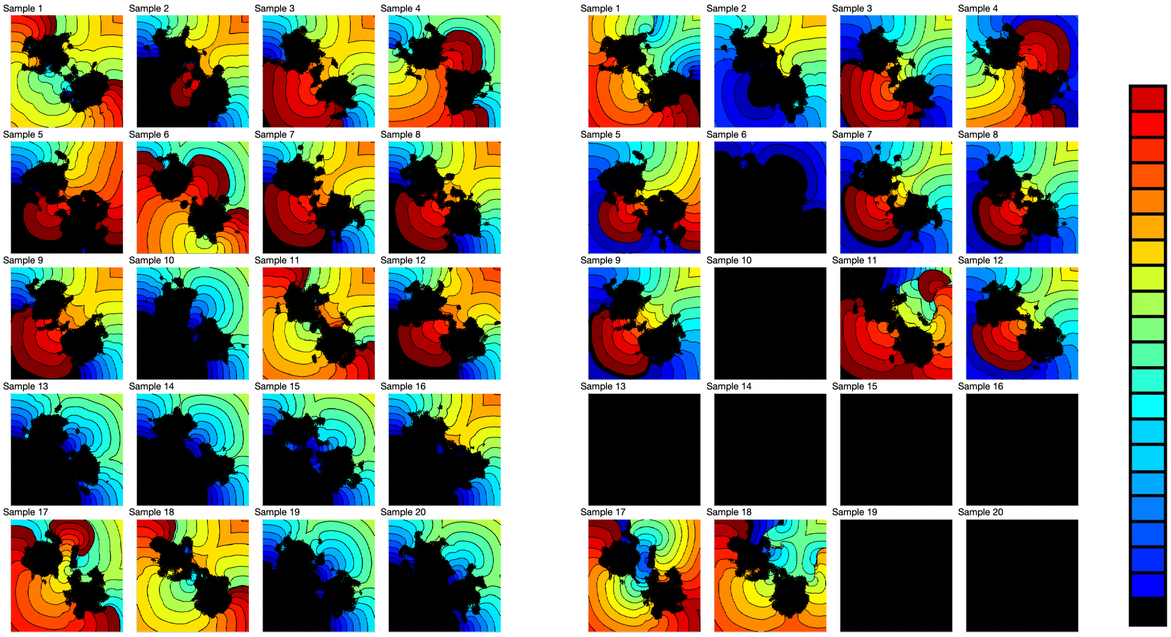

(a) 2200 ms to 2580 ms

(b) 2600 ms to 2980 ms

Figure 23: *ContinuousD-random* model, length scale 5.0 mm.

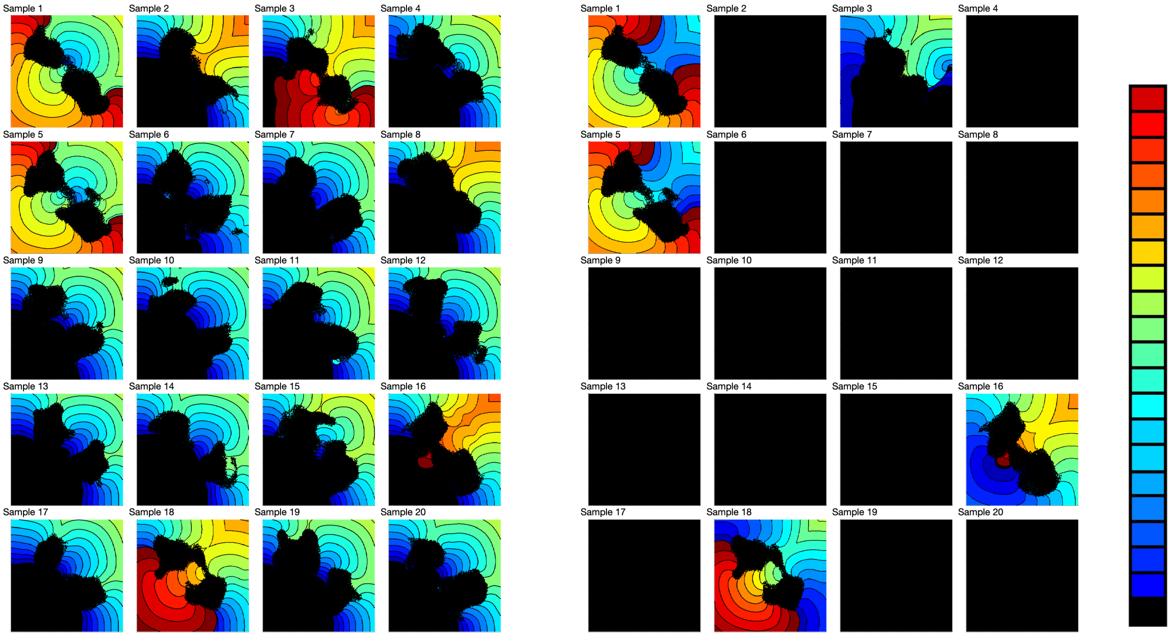

(a) 2200 ms to 2580 ms

(b) 2600 ms to 2980 ms

Figure 24: *ContinuousD-random* model, length scale 10.0 mm.
